# Wearable device and smartphone data quantify ALS progression and may provide novel outcome measures

**DOI:** 10.1038/s41746-023-00778-y

**Published:** 2023-03-06

**Authors:** Stephen A. Johnson, Marta Karas, Katherine M. Burke, Marcin Straczkiewicz, Zoe A. Scheier, Alison P. Clark, Satoshi Iwasaki, Amir Lahav, Amrita S. Iyer, Jukka-Pekka Onnela, James D. Berry

**Affiliations:** 1 Mayo Clinic, Department of Neurology, Scottsdale, AZ USA; 2 Harvard T.H. Chan School of Public Health, Boston, MA USA; 3 Neurological Clinical Research Institute and Sean M. Healey & AMG Center for ALS, Massachusetts General Hospital, Boston, MA USA; 4 Mitsubishi Tanabe Pharma Holdings America, Inc, Jersey City, NJ USA

**Keywords:** Biomarkers, Amyotrophic lateral sclerosis, Amyotrophic lateral sclerosis, Biomarkers

## Abstract

Amyotrophic lateral sclerosis (ALS) therapeutic development has largely relied on staff-administered functional rating scales to determine treatment efficacy. We sought to determine if mobile applications (apps) and wearable devices can be used to quantify ALS disease progression through active (surveys) and passive (sensors) data collection. Forty ambulatory adults with ALS were followed for 6-months. The Beiwe app was used to administer the self-entry ALS functional rating scale-revised (ALSFRS-RSE) and the Rasch Overall ALS Disability Scale (ROADS) surveys every 2–4 weeks. Each participant used a wrist-worn activity monitor (ActiGraph Insight Watch) or an ankle-worn activity monitor (Modus StepWatch) continuously. Wearable device wear and app survey compliance were adequate. ALSFRS-R highly correlated with ALSFRS-RSE. Several wearable data daily physical activity measures demonstrated statistically significant change over time and associations with ALSFRS-RSE and ROADS. Active and passive digital data collection hold promise for novel ALS trial outcome measure development.

## Introduction

Amyotrophic lateral sclerosis (ALS) therapeutic development has largely relied on staff-administered functional rating scales (i.e. ALS functional rating scale-revised – ALSFRS-R) to gauge interventional efficacy in large-scale clinical trials with limited success. Alternative methods of assessing progressive functional decline in ALS may be valuable, such as using mobile applications (apps) to collect patient-reported outcome measures (PROMs; e.g. the self-entry version of the ALSFRS-R – ALSFRS-RSE) and wearable devices for measurement of physical activity (PA).

Several factors contribute to the difficulty in finding efficacious ALS treatments. Although ALS fits well into the motor neuron disease category, it remains a heterogeneous disorder with respect to onset location, the pattern of progression, degree of upper motor neuron involvement, underlying genetic architecture, patient experience, and prognosis^1,2^. It is uncommon in the general population despite being the most common motor neuron disease^3^, has a poorly understood pathobiology^4^, and remains a clinically defined syndrome. Traditional ALS outcome measure limitations may also play a role. Factors outside of ALS itself and germane to all clinical trial research add to the difficulty of understanding whether an intervention works. The need for a substantial research infrastructure, experienced staff, and adequate coordinator support for recruitment and logistical operations are obstacles^5^. Trial visit frequency is designed factoring in staff availability, participant willingness and ability to travel, and cost. While higher trial outcome sampling frequency is typically advantageous, it usually requires additional resources. All these aspects slow progress toward finding a cure.

The ALSFRS-R is a validated, traditionally staff-administered, 12-item, 0–4 points/item scale used to assess function and disease progression over time. It has served as the primary longitudinal ALS clinical trial outcome measure for years;^6–9^ however, the scale has limitations. The ALSFRS-R does not meet the criteria for unidimensionality^8,10^, it is ordinal meaning point-wise decline is nonuniform with each score decrease, and it may fail to detect decline in people with confirmed ALS (PALS) even over the course of 18-months based on Pooled Resource Open-Access ALS Clinical Trials Database (PRO-ACT) data. Over the course of 6-months (the duration of many clinical trials), 25% of participants on placebo or a non-efficacious intervention did not decline in the PRO-ACT dataset^9^.

Though extensive data have been collected to date using traditional functional rating scales, different assessment approaches may have advantages, especially PROMs. Self-entry functional rating scales can reduce the burden of frequent trial center visits for patients and allow home monitoring of disease progression^11^. A self-entry version of the ALSFRS-R, the ALSFRS-RSE, has been developed and has repeatedly demonstrated excellent correlation with the ALSFRS-R (staff-administered)^11–16^. Other scales are being developed using modern test theory techniques and for self-entry. One such example is the Rasch-built Overall ALS Disability Scale (ROADS)^10,17^. Prior studies have demonstrated the feasibility of both in-clinic and non-clinic ALS PROM collection^15,18^.

Remote, passive data collection offers another avenue for outcome measure refinement without the need for participants’ concentration or effort. Modern digital wearable monitors allow for detailed, objective, and near-continuous motor and other biometric measurements^19^. Furthermore, they enable the assessment of new and previously challenging to quantify human functional domains. Digital biomarkers have been used already in the neuromuscular and neurodegenerative disease sphere, with significant expansion expected^20^. Viability of periodic non-clinic device and sensor-based data collection has been demonstrated in measuring heart rate variability, accelerometry-based activity, speech, spirometry, hand-grip strength, and electrical impedance myography^21–24^. Few studies have examined continuous monitoring of PALS using wearable devices. Some have measured 3-day/month samples of PA and heart rate variability using a chest-worn and ECG sensor Faros device^21,23^. Kelly et al. reported four PA level endpoints showing moderate or strong between-patient correlations with ALSFRS-R total score and gross motor domain score^23^. Van Eijk et al. ^22^ employed a hip-worn ActiGraph device for 7 days every 2-3 months for 12 months and reported four accelerometer-based endpoints of PA levels to be significantly associated with the ALSFRS-R.

Mobile devices may also represent a valuable mode of digital biomarker collection. Smartphones are becoming nearly ubiquitous, with 85% of the United States (US) population owning one and over 7 billion smartphones worldwide^25,26^. Smartphone sensors coupled with mobile apps allow for versatile data collection of both active (e.g., surveys, audio recordings) and passive (e.g., global positioning system (GPS), accelerometry) data^27^. They have also shown promise collecting these data longitudinally from PALS^28^.

We evaluated whether an app and wearable devices might serve as important tools for monitoring ALS disease progression through active (app surveys) and passive (wearable device sensors) data collection.

We aimed to (1) compare (smartphone) ALSFRS-RSE with (staff-administered) ALSFRS-R at baseline and longitudinally, (2) quantify the baseline and change over time of wearable daily measures of PA, and (3) quantify the association between those measures and both the ALSFRS-RSE and ROADS total scores.

## Results

### Participants, enrollment, and compliance

From January through December 2021, 46 participants with ALS were enrolled and followed for 6 months. Of these, 40 met analysis sample criteria. One participant was unable to download the Beiwe app. Participants chose their wearable device and did not favor one over the other (ActiGraph = 20, Modus = 20). Clinicodemographic characteristics were similar between device groups (Table 1). Overall, there was a higher proportion of male (62.5%), white (87.5%), and non-Hispanic participants (87.5%). Most participants’ smartphones ran iOS (82.5%). Median baseline ALSFRS-R was 33 (range 11–47). Compliance statistics are summarized in Table 2 and were robust for both groups.

**Table 1:** Baseline demographic and clinical characteristics.

	ActiGraph	Modus	Combined
	*N* = 20	*N* = 20	*N* = 40
Age			
Mean (SD)	62.9 (13.4)	60.6 (10.7)	61.8 (12.0)
Median [min, max]	64 [34, 98]	60 [35, 81]	63 [34, 98]
Sex			
Males (%)	12 (60.0%)	13 (65.0%)	25 (62.5%)
Females (%)	8 (40.0%)	7 (35.0%)	15 (37.5%)
Ethnicity			
Not Hispanic or Latino (%)	18 (90.0%)	17 (85.0%)	35 (87.5%)
Hispanic or Latino (%)	2 (10.0%)	2 (10.0%)	4 (10.0%)
Unknown / Not Reported (%)	0 (0.0%)	1 (5.0%)	1 (2.5%)
Race			
White (%)	17 (85.0%)	18 (90.0%)	35 (87.5%)
More Than One Race (%)	2 (10.0%)	1 (5.0%)	3 (7.5%)
Native Hawaiian or Other Pacific Islander (%)	1 (5.0%)	0 (0.0%)	1 (2.5%)
Other (%)	0 (0.0%)	1 (5.0%)	1 (2.5%)
Phone operating system			
iOS (%)	15 (75.0%)	18 (90.0%)	33 (82.5%)
Android (%)	5 (25.0%)	2 (10.0%)	7 (17.5%)
ALSFRS-R			
Mean (SD)	31.4 (8.6)	31.4 (7.9)	31.4 (8.1)
Median [min, max]	33 [12, 47]	32 [11, 45]	33 [11, 47]

*ALSFRS-R* baseline staff-administered Amyotrophic Lateral Sclerosis Functional Rating Scale-Revised, *n* number, *min* minimum, *max* maximum, *SD* standard deviation.

**Table 2:** Phone-survey and wearable device compliance (Aggregate -- median [min, max]).

	ActiGraph	Modus	Combined
ALSFRS-RSE submissions	5 [2, 10]	6 [2, 13]	5 [2, 13]
ROADS submissions	4 [2, 10]	5 [2, 13]	5 [2, 13]
Days in observation period	178 [23, 191]	180 [61, 189]	179 [23, 191]
Valid days in observation period*	158 [21, 191]	136 [16, 183]	146 [16, 191]
Average number of valid hours on a valid day*	21 [15, 24]	12 [10, 17]	16 [10, 24]

*ALSFRS-RSE* smartphone self-entry Amyotrophic Lateral Sclerosis Functional Rating Scale-Revised, *ROADS* Rasch-built Overall ALS Disability Scale; valid hours/day -- hours/day passing the conditions outlined in Methods; * -- different methods used to define valid hour/day between the ActiGraph and Modus groups (see Methods section).

### Correlation between ALSFRS-R and ALSFRS-RSE

**Fig. 1:**
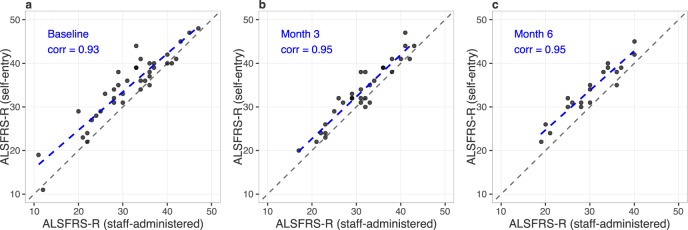
Correlation of ALSFRS-R (staff-administered) and (smartphone) self-entry ALSFRS-R. Baseline (**a**), 3-month (**b**), and 6-month (**c**) correlations. The blue dashed line represents the observed linear association. The black dashed line is a 45-degree line.

Smartphone self-administered ALSFRS-RSE was highly correlated with staff-administered ALSFRS-R at baseline, 3-, and 6-months (*r* ≥ 0.93; Fig. 1). The population mean for ALSFRS-RSE was higher than that of ALSFRS-R at each time point (by 3.3, 2.2, and 3.7 points, respectively).

### Survey baselines and change over time

**Fig. 2:**
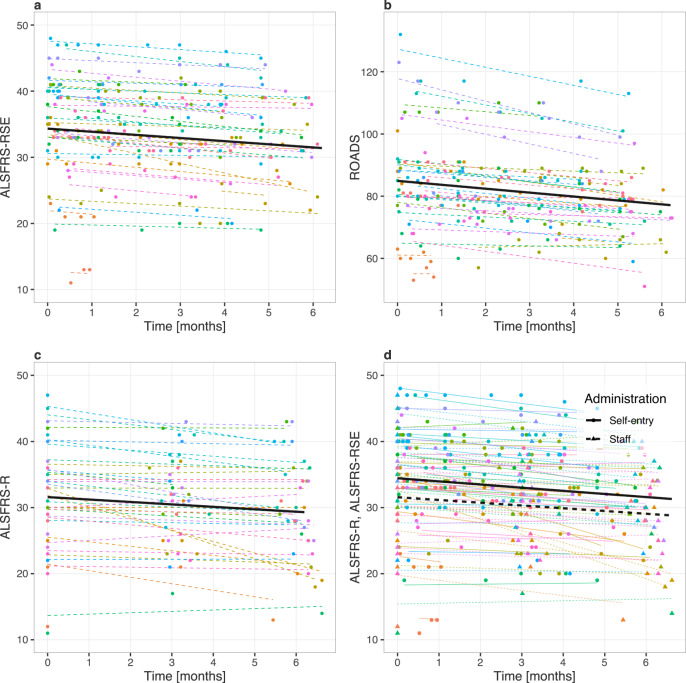
Estimated survey baseline and change over time. **a** Self-entry Amyotrophic Lateral Sclerosis Functional Rating Scale-Revised (ALSFRS-RSE). **b** Rasch-built Overall ALS Disability Scale (ROADS). **c** Staff-administered Amyotrophic Lateral Sclerosis Functional Rating Scale-Revised (ALSFRS-R). **d** Combined ALSFRS-RSE and ALSFRS-R plots. Participants are color coded and the color scheme is maintained across plots: lines represent participant-specific conditional means, points represent observed values. The thick black lines in **a**, **b**, and **c** represent the population means. In **d**, the population baselines and slopes are estimated for both the ALSFRS-R (staff-administered) and ALSFRS-RSE (self-entry) values in a single regression model.

Mean baseline scores were 31.6 (ALSFRS-R), 34.3 (ALSFRS-RSE) (max 44, higher is better), and 84.9 (ROADS) (max 146, higher is better); and monthly rates of decline ([95% CI], p-value) were −0.37 ([-0.62,−0.11], 0.007), −0.48 ([−0.63,−0.32], 0.001), and −1.26 ([−1.71,−0.81], <0.001), respectively (Fig. 2a, b, c; Supplementary Table 1, models 1–3). The ALSFRS-RSE baseline was 2.86 points higher than the ALSFRS-R baseline score (95% CI [2.26,3.47]; *p* < 0.001), but the monthly change in slope between the scales did not significantly differ (Fig. 2d; Supplementary Table 1, model 4).

Comparing ActiGraph and Modus groups, there was no significant difference in baseline or monthly change in either ALSFRS-RSE or ROADS (Supplementary Fig. 1a and b; Supplementary Table 1, models 5 and 6).

### Wearable daily Physical Activity (PA) measures: baseline values and a change over time

Table 3 summarizes the average baseline and monthly change of wearable daily PA measures. Estimates are provided for 13 ActiGraph vendor-provided daily (24-hour period) summary measures (VDMs), 7 ActiGraph investigator-derived daily summary measures (IDMs), and 12 Modus VDMs. 23 out of 32 measures demonstrated significant change over time, e.g., total activity counts (ActiGraph VDM) -56,625/month, sedentary minutes (ActiGraph IDM) + 13/month, total steps (Modus VDM) -58/month. All physical activity volume measures declined, except sedentary minutes (ActiGraph VDM; non-significant effect) and non-locomotion minutes (ActiGraph VDM; significant effect). Participants’ individual conditional means and the population mean for each measure are depicted (Supplementary Figs. 2–12, first columns). The following measures had a significant and at least 10% relative change from baseline in at least 80% of participants over 6 months based on conditional means: steps (ActiGraph VDM), sedentary to active transition probability (ActiGraph IDM), total steps (Modus VDM), % time with 16-40 steps recorded/minute (Modus VDM) (Table 1). The following significant measures had very high (≥ 0.85) conditional coefficient of determination (R2c) values: log total activity counts (ActiGraph VDM), active to sedentary transition probability (ActiGraph IDM), and mean steps/minute (Modus VDM)^29,30^. Total activity counts (TAC) are the sum of activity counts (AC) over a period of time (here, 24 hours). AC are derived from raw accelerometer data and are a unitless measure that quantifies acceleration signal magnitude within a time interval (here, 60 seconds).

**Table 3:** Average baseline and monthly change in wearable daily physical activity measures.

Mod.	Device	Data	Daily measure	Baseline est. [95% CI]	Monthly change	R2m	R2c	% with 6 m rel.
no.	group	set			est. [95% CI] (p-val.)			change ≥ 10%
1	AG	VDMs	Light activity [minutes]	1283 [1217, 1349]	−24.75 [−48.29, −1.215] (0.040)	0.033	0.459	30%
2	AG	VDMs	Moderate activity [minutes]	51.12 [35.00, 67.25]	−0.581 [−3.949, 2.788] (0.719)	0.000	0.585	70%
3	AG	VDMs	Vigorous activity [minutes]	4.032 [-1.130, 9.194]	−0.069 [−0.363, 0.226] (0.643)	0.000	0.507	90%
4	AG	VDMs	MVPA [minutes]	55.17 [36.28, 74.06]	−0.691 [−4.201, 2.818] (0.682)	0.000	0.647	70%
5	AG	VDMs	Sedentary [minutes]	1058 [987.3, 1130]	−13.06 [−32.25, 6.122] (0.170)	0.009	0.560	30%
6	AG	VDMs	Nonsedentary [minutes]	279.7 [204.5, 354.9]	−13.08 [−22.50, -3.662] (0.010)	0.022	0.819	75%
7	AG	VDMs	Locomotion [minutes]	23.17 [10.23, 36.12]	−0.526 [−2.143, 1.091] (0.502)	0.001	0.772	80%
8	AG	VDMs	Nonlocomotion [minutes]	1315 [1250, 1380]	−24.80 [−48.39, −1.219] (0.040)	0.033	0.464	30%
9	AG	VDMs	Steps	2661 [760.1, 4561]	−165.7 [−321.1, −10.21] (0.038)	0.005	0.791	80%
10	AG	VDMs	Calories	2109 [1840, 2377]	−53.95 [−100.8, −7.073] (0.027)	0.023	0.772	45%
11	AG	VDMs	METs	1525 [1354, 1697]	−38.42 [−71.32, −5.525] (0.025)	0.033	0.663	45%
12	AG	VDMs	Total activity counts	1292056 [988272, 1595841]	−56625 [−100107, −13144] (0.014)	0.025	0.798	65%
13	AG	VDMs	Sleep [minutes]	659.5 [538.4, 780.7]	−1.691 [−31.44, 28.06] (0.905)	0.000	0.558	15%
14	AG	IDMs	Total activity counts	1362438 [1046388, 1678489]	−58631 [−102202, −15059] (0.012)	0.025	0.824	65%
15	AG	IDMs	Total log(activity counts)	5456 [4808, 6104]	−127.7 [−236.9, −18.47] (0.026)	0.029	0.707	65%
16	AG	IDMs	log(total activity counts)	13.90 [13.50, 14.31]	−0.046 [−0.078, −0.013] (0.011)	0.009	0.934	0%
17	AG	IDMs	Active to sedentary TP	0.389 [0.299, 0.479]	0.009 [0.003, 0.014] (0.003)	0.005	0.890	60%
18	AG	IDMs	Sedentary to active TP	0.074 [0.057, 0.091]	−0.003 [−0.006, −0.000] (0.028)	0.026	0.723	80%
19	AG	IDMs	Nonsedentary [minutes]	271.4 [200.6, 342.2]	−12.88 [−22.23, −3.530] (0.010)	0.023	0.809	70%
20	AG	IDMs	Sedentary [minutes]	1169 [1098, 1239]	12.88 [3.530, 22.23] (0.010)	0.023	0.809	25%
21	M	VDMs	Total steps	1871 [1137, 2605]	−57.79 [−89.26, −26.32] (0.001)	0.003	0.836	80%
22	M	VDMs	Steps recorded/minute - 95th perc.	25.92 [21.19, 30.65]	−0.533 [−1.015, −0.052] (0.032)	0.005	0.734	40%
23	M	VDMs	Steps recorded/minute - mean	9.170 [7.050, 11.29]	−0.149 [−0.265, −0.034] (0.015)	0.003	0.858	45%
24	M	VDMs	Steps recorded/minute - median	6.793 [4.566, 9.020]	−0.081 [−0.183, 0.020] (0.110)	0.001	0.845	30%
25	M	VDMs	Percent time with 1-15 steps recorded/minute	9.395 [7.334, 11.46]	−0.187 [−0.315, −0.060] (0.006)	0.004	0.731	55%
26	M	VDMs	Percent time with 16-40 steps recorded/minute	2.743 [1.451, 4.036]	−0.120 [−0.182, −0.057] (0.001)	0.005	0.810	90%
27	M	VDMs	Percent time with 41+ steps recorded/minute	0.293 [0.026, 0.560]	−0.001 [−0.012, 0.010] (0.863)	0.000	0.754	70%
28	M	VDMs	Max 60	8.776 [5.947, 11.61]	−0.256 [−0.502, −0.009] (0.043)	0.003	0.708	75%
29	M	VDMs	Max 20	14.89 [10.60, 19.18]	−0.424 [−0.904, 0.057] (0.081)	0.003	0.669	65%
30	M	VDMs	Max 5	24.55 [18.98, 30.11]	−0.736 [−1.276, −0.195] (0.011)	0.007	0.729	60%
31	M	VDMs	Max 1	35.56 [29.71, 41.41]	−0.785 [−1.374, −0.196] (0.012)	0.007	0.793	40%
32	M	VDMs	Peak performance index	23.21 [17.92, 28.49]	−0.593 [−1.017, −0.170] (0.009)	0.005	0.810	50%

*AG* ActiGraph device, *CI* estimate’s confidence interval obtained from LMM estimation, *est*. estimate obtained from LMM estimation, *IDMs* investigator-derived daily measures, *M* Modus device, *Max* (60,20,5,1) – maximum consecutive cadence during (60,20,5,1) minutes, *Mod. no*. model number: ordering index of a LMM fit, assigned to a particular daily measure, *MVPA* moderate and vigorous physical activity, *PPI* mean cadence of the day’s most intensive, noncontiguous 30 minutes, *R2c* LMM conditional coefficient of determination, *R2m* LMM marginal coefficient of determination; *VDMs* vendor-provided daily measures; % with 6 m rel. change > =10% -- the percentage of participants for whom LMM-fit conditional means changed ≥ 10% from baseline and in the same direction as the population-level change over 6 months.

### Wearable daily Physical Activity (PA) measures: association with both ALSFRS-RSE and ROADS

The mixed-effects models (LMM) associations between each daily measure (VDMs,IDMs) and each survey (ALSFRS-RSE, ROADS) are summarized (Supplementary Tables 1-3) and graphically depicted (Supplementary Figs. 2-12, 2nd and 3rd columns). The daily measures were the covariates in the LMM and were standardized to have means equal to 0 and standard deviations equal to 1. 13/32 daily measures were significantly associated with both ALSFRS-RSE and ROADS, which included the following ActiGraph VDMs: total activity counts, moderate activity, moderate and vigorous physical activity, non-sedentary minutes, locomotion minutes, steps, calories, as well as the following ActiGraph IDMs: total activity counts, log total activity counts, active to sedentary transition probability, sedentary to active transition probability, sedentary and non-sedentary minutes. None of the Modus measures were significantly associated with both surveys. Overall, lower ALSFRS-RSE and ROADS scores were associated with measures reflecting lower PA volume, and lower sedentary to active/higher active to sedentary transition probabilities (activity fragmentation measures).

### Sensitivity analysis of wearable device monitoring frequency

Using all weeks’ (6-months) data, the estimated baseline total activity counts (ActiGraph IDM) were 1,362,438 and the monthly change was -58,631. Across the 9 different data collection frequency scenarios examined (Supplementary Table 4), LMM results did not vary substantially (≤1.5% change) from the baseline estimate using all available data. The monthly change estimate did not vary substantially (<4%) for scenarios with 2 weeks of data collection and up to 6 weeks break but did (9.2-24.5%) for the others compared to using all weeks’ data. All scenarios detected significant decline in total activity counts over time.

## Discussion

Forty ambulatory adults with ALS were followed for 6 months during which they continuously wore a wearable device (the wrist-worn ActiGraph’s Insight Watch or the ankle-worn Modus’ StepWatch) and regularly contributed ALSFRS-RSE and ROADS surveys via the Beiwe smartphone app. Daily measures of physical activity (PA) from the wearables allowed quantification of baseline values, monthly PA volume decline, and other characteristics.

Though ambulatory status was an inclusion criterion, PALS with a range of functional abilities (baseline ALSFRS-R ranged from 11 to 47) met this criterion, increasing generalizability. Given that PALS frequently have limb weakness and fatigue affecting activity levels, 8 hours of activity logged per day was chosen as the daily device wear compliance threshold. The number of compliant days and active hours per day differing between the ActiGraph and Modus groups should not be interpreted as meaningfully different because the devices collected data differently, thus methodologies used for valid hour determination differed. Despite the observational nature of the study, older age group, and use of multiple technologies, device wear- and survey submission-compliance were robust in both device groups. Compliance is often quite variable in many digital health studies, both within and between studies: levels range from 25-80%^31–34^. The number of Beiwe app survey submissions was much higher than the number of assessments typically obtained in traditional in-person clinical trials (Table 2)^24^.

Excellent correlation between staff-administered ALSFRS-R and smartphone self-administered ALSFRS-R ( ≥ 0.93), along with higher (2.86 points) self-report baseline scores, are consistent with prior studies^11,15,16^, reinforcing that valuable functional outcome data can be reliably obtained remotely and that the ALSFRS-R, the mainstay of outcome assessment in ALS trials, can be reliably obtained in this manner.

The significant difference between ALSFRS-R and ALSFRS-RSE at baseline but not over time suggests that self-report and staff-administered scores should be compared separately to avoid introducing unwanted variability, something to be considered during trial design.

In general, all three scales (ALSFRS-R, ALSFRS-RSE, ROADS) declined as expected and performed well in detecting functional decline despite our participants having a milder rate of decline as a group compared to those in interventional trials^35–38^. Observational studies tend to enroll PALS with more slowly progressing disease^22,24^.

Notably, neither ActiGraph nor Modus VDMs explicitly account for missing data due to non-wear, charging, etc. For example, ActiGraph’s non-sedentary and sedentary minutes add up to 1440 minutes for IDM but do not for VDM. Consequently, PA volume and performance intensity measures may be biased downward. To explore what results might show if accounting for missing data, we created a set of IDMs that imputed missing minute-level data. As expected, the baseline total activity counts were lower for VDM compared to IDM (1,292,056 vs 1,362,438). The IDMs we employed utilize straightforward code (link in Methods), thus this work can be reproduced, built upon, and scaled relatively easily. The IDMs also had much stronger associations and were statistically significant. These findings also suggest there may be other, even better, measures for detecting functional decline. Similarly, in a Nuedexta study, the effects of the drug on speech features were not detected by speech-language pathologists but were detected using objective measures^39^.

The model estimation process converged for all LMM discussed. The marginal coefficient of determination (R2m) represents the proportion of the variance explained by fixed effects and R2c represents the proportion of the variance explained by both fixed and random effects.

The majority of the wearable measures significantly changed over time. We calculated R2c, the proportion of the variance explained by both fixed and random effects, to identify measures with very high R2c ( ≥ 0.85). Measures of PA volume and fragmentation had very high R2c and were well characterized with the use of relatively simple LMMs: log total activity counts, active to sedentary transition probability (ActiGraph IDM), and mean steps/minute (Modus VDM).

The 13 wearable physical activity (PA) measures with significant associations with both surveys (ALSFRS-RSE and ROADS) centered around higher PA volume. This suggests that the specific type of PA monitoring may not matter as much as the volume of activity being monitored. There appeared to be a trend towards more intense activity. ALS, which is progressive, makes it harder for participants to achieve vigorous activity levels. This led to less high-intensity data being available for analysis and may be why more measures did not meet criteria. Our use of a stringent definition requiring significant associations with both ALSFRS-RSE and ROADS also reduced the number of positive measures. It should be noted that this study was not designed to assess whether one device more accurately quantifies physical change over time than the other and such conclusions cannot be drawn from the results. Further exploration with larger data sets is needed to determine whether different levels of physical activity intensity are better suited to detecting functional decline, find the most sensitive measures, refine derivation methods, establish clinically meaningful thresholds, and so on.

Results obtained in the sensitivity experiment comparing different scenarios of data collection protocols indicated that for total activity counts (ActiGraph IDM; R2c 0.82), as long as 2 weeks of data were collected, there was no substantial change in the baseline value or monthly change value with a break in data collection up to 6 weeks. Change in slope varied substantially in the less frequent monitoring scenarios (up to 24.5%) but all data collection frequencies found significant functional decline associations, even 1 week of data with 8 week breaks in between (1wD + 8wB). This is a very important finding, as it (1) points to the ability to identify thresholds of data collection needed to accurately track functional decline and (2) shows that days-long continuous data collection may be an adequate substitute for months-long data collection in some cases. This analysis highlights the conceptual challenge of new metrics and the need for ensuring clinical relevance by evaluating what constitutes clinically meaningful change. Given that -58,600 total activity counts/month was the observed rate of decline among the participants, who were also declining on validated functional rating scales, this rate of decline is clinically meaningful, though specific cutoffs have yet to be defined.

These findings affirm the feasibility and utility of remote monitoring with the use of apps and wearable devices for the collection of PROMS and daily measures of physical activity in PALS.

Remote/at-home digital biometric data collection has several benefits for the field, which make it attractive for outcome measure development. First, it enables more frequent sampling, which means that trials need not enroll as many participants to know whether an intervention is efficacious. This accelerates trial results and is also important in relatively uncommon diseases where recruitment may be difficult. Second, remote digital biometric data collection decreases participant burden, especially important in a population that experiences mobility challenges. Third, it may increase access and inclusion, allowing those who are geographically or socioeconomically isolated to participate. Fourth, there is likely benefit to assessing characteristics of daily life activities not captured by, or which have been out of reach, of functional rating scales, such as measuring one’s step count or any one of several features by which gait can be quantified and analyzed (e.g. cadence). Variables that don’t lend well to self-report or clinical assessment but which may have meaningful effects on health, such as active to sedentary transition probability, can now be captured. Fifth, digital data collection can reduce changes in performance that might occur during an in-person visit when one knows s/he is being assessed, quantify function in one’s lived-in environment, and eliminate interrater differences. Sixth, objectively measured physical function data could be valuable, especially in a disease that profoundly affects quality of life and for which we rely on patient responses to generate metrics. Seventh, digital technology and data can also be highly accessible to participants, staff, and other researchers. Depending on the technology and vendors, it can be low-cost and quite scalable^40^. Finally, remote outcome assessment can reduce participants’ exposure risk to pathogens (i.e. COVID-19).

There are challenges with remote, digital data collection, some of which may be unexpected. For example, we found that one participant’s partner often carried her phone on his person. One participant downloaded the Beiwe app onto a tablet rather than a smartphone, despite instruction. One participant using the Modus wearable routinely used a stationary desk-cycle. These examples highlight how isolated metrics in the absence of context can be difficult to interpret and vigilance is needed to make sure that measures are meaningful and evaluating as intended. Depending on the study, managing the high data volumes from passive monitoring can be logistically difficult for the device, participant, and/or researchers. Device feature design is of paramount importance, especially in a population that frequently loses hand strength and dexterity. Participants can change the side on which they wear the device. Compliance is frequently an issue, despite the fact that research participants are likely more adherent than non-research patients. Technological difficulties, such as how to use the app (log back in, resolve glitches, etc.) all affect data collection and can require study coordinator support. In addition, some participants, experienced high battery drain, which they found bothersome. Usability factors can be anticipated, such as designing straps and devices so that PALS with hand weakness can utilize them without undue burden. The advent of data sharing and open-source code enables a collaborative, transparent research environment. Caution is also needed to ensure systems perform as expected and that participant privacy is protected^41^.

Until there is a cure for ALS, we must work to better understand the disease and improve our efficiency at investigating potential therapeutics. Having the best diagnostic and monitoring biomarkers and outcome measures to quantify disease progression are key. Just as ALS diagnostic research criteria have iteratively evolved over time^42–45^, so too, should our outcome measures if there is room for improvement. The COVID-19 pandemic has shown us that there are opportunities for enhanced research and care through technology incorporation^46,47^.

This work generates many avenues for future directions in trial application validation, novel outcome measure development, and existing PROM and digital outcome measure refinement. Understanding how data is affected by the use of various assistive devices is also important. Ultimately, incorporation of digital outcome measures into interventional clinical trials alongside standard functional assessments will help clarify their role in boosting trial efficiency and acting as key outcome measures. Whether they can enhance versus supplant current outcome measures and assessment methods remains to be seen, and may require multidisciplinary development to harness other technologies such as artificial intelligence.

We did not have a healthy control arm to which to compare results. There was missing data, resulting from imperfect compliance, though not enough to substantively affect data integrity. It is a reminder, however, that digital interface design, beta testing, and robust support for technical issues are critically important for ensuring data quality in digital studies. The study enrolled a more slowly progressing population than interventional trials, thus testing digital biomarkers in trial populations should be performed. Our ethnoracial diversity was low, but more inclusive than most US ALS studies. This may suggest that, properly applied, digital technology can bridge the gap between underrepresented populations in ALS research.

This study demonstrated in a non-rapidly progressing, functionally heterogeneous group of PALS that passively collected sensor data from wearable devices (either wrist-worn or ankle-worn) can characterize daily physical activity and its change over time. These measures were also found to be significantly associated with ALSFRS-RSE and ROADS scores. Evaluation of digital outcome measures in interventional clinical trials is needed to understand whether they may have a role as primary outcome measures and enhance clinical trial efficiency.

## Methods

### Ethics

Massachusetts General Brigham (MGB) Institutional Review Board (IRB) and Massachusetts General Hospital (MGH) Information Security Office approvals were obtained prior to study initiation. All data were collected and maintained in accordance with MGB, state, and national policies and regulations. Participants provided written informed consent prior to participation in study procedures, and we complied with all relevant ethical regulations.

### Study design and population

This was a single-center, non-interventional, remote study enrolling ambulatory participants over the age of 18 years with a diagnosis of ALS by El-Escorial Criteria^44^ who were able to provide informed consent, comply with study procedures, and operate their smartphone without assistance – as determined by the site investigator’s assessment. The study was advertised on ALS social media accounts, an institutional research recruitment website, and to patients attending the ALS multidisciplinary clinic. Study procedures were performed remotely, with rare exception (participant on site). Participants were observed for 6-months and received $50 at 3- and 6-months if still contributing data.

### Data collection and devices

**Fig. 3:**
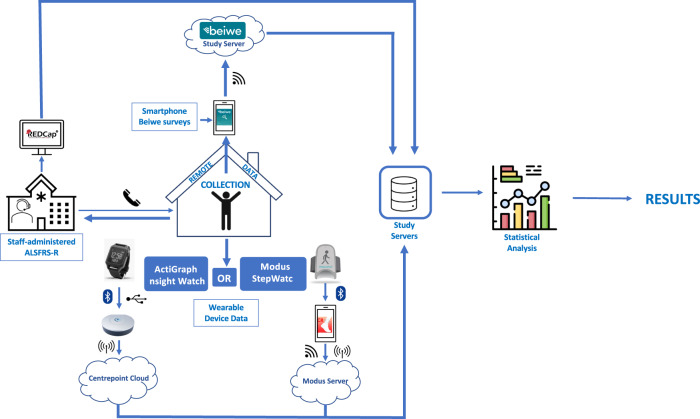
Study data flow schematic. Each participant provided three data streams (blue text and white background thin-outline boxes). Thick blue arrows represent directionality of data. The small icons adjacent to the blue arrows represent the mode of data transfer: vertical oval with blue background and white lines (Bluetooth); dot with three curved lines (Wi-Fi); phone (study staff-initiated phone call); vertical line with curved vertical lines on either side (cellular). ALSFRS-R – Amyotrophic Lateral Sclerosis Functional Rating Scale-Revised.

Data were collected simultaneously in three ways: (1) staff-administered ALSFRS-R surveys by phone/televisit, (2) remote survey completion using the Beiwe app, (3) and wearable device passive data collection (Fig. 3).

Following consent, study staff screened participants, and, if eligible, performed baseline visit procedures: demographics, baseline ALSFRS-R, download of and instruction on the use of the Beiwe platform (Beiwe) app on his/her smartphone (iOS or Android), and wearable device selection. Participants were called after receipt of their chosen device to review proper use and maintenance.

Beiwe is an open-source digital phenotyping research platform designed specifically for the collection of research-grade, raw (unprocessed) data from smartphone sensors, logs and self-entry measures (surveys, voice recordings)^48^. Data collected with the Beiwe app are encrypted and stored on the smartphone until Wi-Fi transfer to the HIPAA-compliant Amazon Web Services (AWS) cloud occurs. Data remains encrypted at each stage.

The app was configured to administer the ALSFRS-RSE and ROADS surveys at baseline and every 2-4 weeks. Contacting participants was permitted if it seemed they were having technical difficulties or asked for help, though these calls were not systematically logged. The app was removed following study completion.

Two wearable devices were used: a wrist-worn ActiGraph Insight Watch (ActiGraph) purchased from ActiGraph LLC and an ankle-worn StepWatch 4 (Modus) from Modus Health LLC. Participants were instructed to wear their device as much as possible (preferably 24 h); Modus participants were told they could remove it during sleep. Participants were not instructed to wear their device on a specific side. Devices were returned using prepaid, pre-addressed packaging.

The ActiGraph Insight Watch is an activity monitor equipped with a triaxial-accelerometer configured to collect raw accelerometry data at 32 Hz. The ActiGraph CentrePoint Data Hub securely transmits de-identified data linked by subject ID using cellular data transfer to the CentrePoint cloud (Fig. 3).

The Modus StepWatch 4 uses a custom mechanical biaxial accelerometer measuring acceleration at 128 Hz. A proprietary algorithm determines if a step was taken and records the corresponding timestamp. If no step is taken, it records that epoch as having 0 steps. It has been validated in various patient populations, including those with impaired gait^49^. This study used Modus’ Clinical Research Trials app software. The device securely transmits de-identified data linked by a subject ID via Bluetooth to the Modus app on participants’ smartphones, which then transmits to Modus’ server using cellular data or Wi-Fi.

Vendor-provided data for both devices were accessible through secure, permissions-based Web Portals. Raw data were provided upon request.

### Wearable daily PA measures

The ActiGraph vendor provided the following processed data: (1) “raw” data -- subsecond-level acceleration measurements; (2) minute-level data: activity counts (AC) -- derived from “raw” data with the open-source algorithm^50^, (x-, y-, and z-) axis counts, steps, calories, metabolic equivalents (METs), wear status, awake status; (3) vendor-provided daily summary measures (VDMs) of time spent: awake and asleep; sedentary and non-sedentary; in locomotion and non-locomotion; in light (<3 METs), moderate (3 to < 6 METs), vigorous (>6 METs), and moderate-to-vigorous PA intensity (MVPA); and the sum of each of the minute-level data measures. VDMs filtered for awake, wear, and awake + wear status were provided. Wear-filtered VDMs (those calculated from periods when the device was worn) were used in our analyses. ActiGraph algorithmically determines wear status from minute-level data, allowing one to ascertain if a device was being worn^51^. Published algorithms are used for calculation of calories and METs^52^ and most of the remaining VDMs^53^. VDMs do not explicitly account for missing data from sensor non-wear or interruptions of raw accelerometry data during device charging and/or data upload. As a result, VDMs may underestimate PA volume^54^.

Using ActiGraph minute-level AC, we created a set of investigator-derived daily measures (IDMs). Minute-level missing AC data was imputed prior to IDM calculation using a participant’s wear-days’ corresponding minutes’ mean AC^54^. IDMs included: total activity counts (24-hour AC sum), log total activity counts (logarithmic transformation of total activity counts + 1), total log activity counts (24-hour sum of logarithmic transformation of AC + 1)^55^, minutes spent active (minutes with AC > 1853)^56^ and inactive, active to sedentary transition probability (fragmentation measure representing the conditional probability a given minute is sedentary given a previously active minute)^57^, and sedentary to active transition probability (conditional probability a given minute is active given a previously sedentary minute)^57^. These measures were chosen given that they have been previously employed in the literature^54–57^, providing the opportunity for comparison and building upon prior work. Data imputation was performed to obtain a data set that more closely represents actual participant activity than one with data missingness.

The Modus vendor provided the following processed data: (1) second-level step count data; (2) minute-level step sums; (3) daily-level (VDM) step counts; percent time in low (1-15 steps/minute), medium (16-40 steps/minute), and high (41+ steps/minute) activity; mean, median, 95th percentile, peak performance index, and max consecutive (60, 20, 5, and 1 minute) cadences. Cadence is defined as steps/minute and does not signify that there was activity during the entire given minute. Peak performance index is the mean cadence of the day’s most intensive, non-contiguous 30 minutes. Modus vendor data did not have an epoch-based wear status indicator; however, a day-level wear status indicator was provided, ascertained through step timestamp examination.

### Data analysis sample

The analysis sample consisted of participants with at least two fully completed ALSFRS-RSE and ROADS surveys, used normed ROADS scores^10^, and used only “valid days” for wearable data (VDMs and IDMs). Valid days were defined as days with at least 8, not necessarily consecutive, “valid hours.” Due to device differences, valid hours were defined uniquely for each wearable. For ActiGraph, a valid hour was defined as 60 consecutive minutes without missing data and vendor-provided wear status indicating device wear. For Modus, a valid hour was one with at least one step logged. This threshold was chosen given that higher thresholds gave very similar results, people with ALS often are less mobile and may have long periods of inactivity, Modus has been shown to have high accuracy in recording observed steps in gait-impaired individuals^49^, and because we did not have raw accelerometry data on which to base minute-increment wear assessment.

### Statistical data analysis

The number of complete ALSFRS-RSE and ROADS submissions were computed for each participant and then aggregated (median and range) across participants by device type (ActiGraph, Modus) and both groups combined. To characterize device wear compliance, the number of days in the observation period, valid days, and average valid hours on a valid day were computed and aggregated.

To quantify ALSFRS-RSE, ALSFRS-R, and their differences at baseline and longitudinally, four LMMs were fitted. Each model assumed time as a fixed effect, participant-specific random intercept and random slope, and differed in outcome (surveys’ total scores): (1) ALSFRS-RSE, (2) ROADS, (3) ALSFRS-R, and (4) ALSFRS-RSE and ALSFRS-R values. Model 4 included an indicator term for the ALSFRS-RSE outcome and a term for the indicator’s interaction with time. To investigate whether differences exist between the ActiGraph and Modus groups’ survey baselines and change over time, two additional LMMs were fitted using time as a fixed effect, participant-specific random intercept and random slope, an indicator for Modus users, and the interaction between the Modus indicator and time with outcomes as ALSFRS-RSE (5), and ROADS (6). In all models (1-6), the time variable was defined as participant-specific elapsed time (in months) from the beginning of the observation period (coinciding with the ALSFRS-R baseline date).

ALSFRS-R and ALSFRS-RSE Pearson’s correlation coefficients (*r*) were calculated at each ALSFRS-R administration: baseline, 3-, and 6-months using participants’ closest matching ALSFRS-RSE within + /− 28 days.

To estimate the average baseline values and the change over time of the wearable daily PA measures, LMMs were fitted separately for each VDM and IDM. Each of the 32 LMM had a daily measure as the outcome, time as a fixed effect, and participant-specific random intercept and random slope. Using participant-specific conditional means, for each measure, we calculated the percentage of participants for whom we observed at least a 10% relative change of the measure over 6 months and for whom the direction of change was consistent with population-level change.

To quantify the association between wearable daily PA measures and ALSFRS-RSE and ROADS scores, LMMs were fitted for each VDM and IDM (32 unique measures) to both ALSFRS-RSE and ROADS separately. The covariates (daily measures) were set as fixed effects, participant-specific random intercept and random slopes were used, and the ALSFRS-RSE/ROADS scores were the outcomes. Covariates were constructed by taking the average of the daily measure’s values spanning the 7 days before and after the survey date for a given participant and survey. To facilitate model comparison, covariates were standardized to have zero means and unit standard deviations.

R2c and R2m for generalized LMMs are reported^29,30^.

A sensitivity analysis was performed to understand how less frequent monitoring might affect the estimated baseline and change over time for the total activity counts (ActiGraph IDM) daily measure. A series of models with different monitoring durations were fitted, each using total activity counts as the outcome, time as a fixed effect, and participant-specific random intercepts and random slopes: all wD (weeks data), 2wD + 2wB (weeks break), 2wD + 4wB, 2wD + 6wB, 2wD + 8wB, 1wD + 2wB, 1wD + 4wB, 1wD + 6wB, and 1wD + 8wB.

All analyses were performed using R software (version 4.2.0; The R Project). R code for all data preprocessing and data analysis is publicly available on GitHub repository (https://github.com/onnela-lab/als-wearables)^58^.

### Reporting summary

Further information on research design is available in the Nature Research Reporting Summary linked to this article.

## Supplementary information

Supplemental materials

REPORTING SUMMARY

## Data Availability

The de-identified data that support the findings of this study are available from the corresponding author upon reasonable request.

## Code availability

R code for all data preprocessing and data analysis is publicly available on GitHub repository (https://github.com/onnela-lab/als-wearables)^58^.
