# Supplementary material for: Wearable device and smartphone data quantify ALS progression and may provide novel outcome measures
Source: NPJ Digit Med. 2023 Mar 6;6:34. doi: 10.1038/s41746-023-00778-y (PMC9987377; doi:10.1038/s41746-023-00778-y)
Supplement: Supplementary file 1 — Supplemental materials [file 41746_2023_778_MOESM1_ESM.pdf]

Supplementary Information

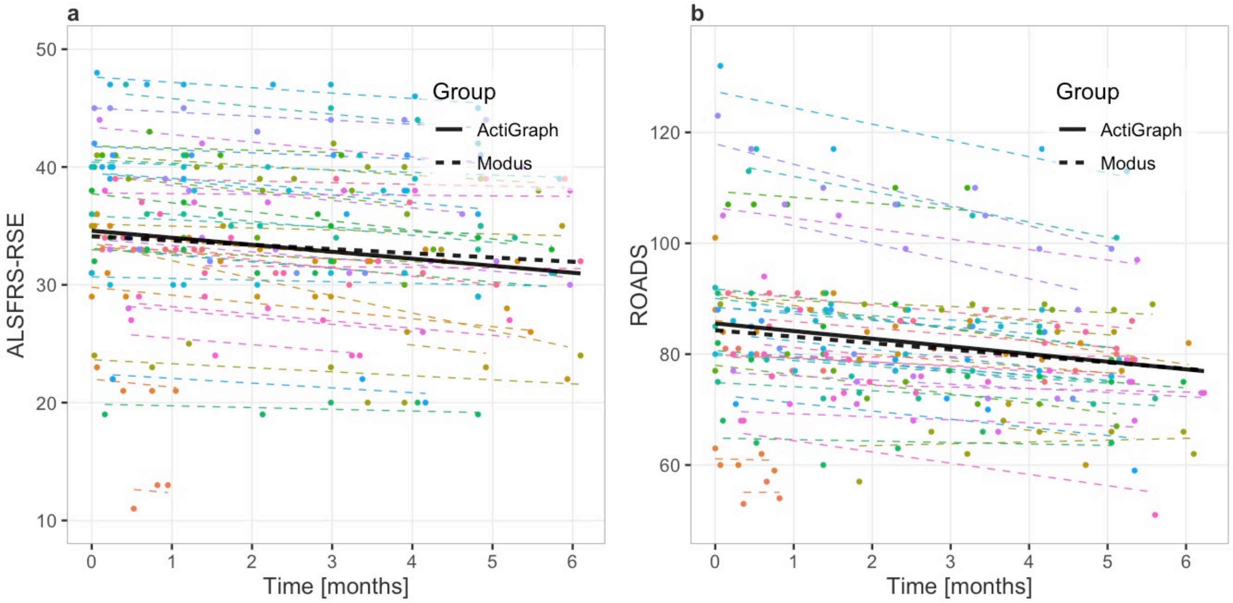

Supplementary Figure 1. **Estimated survey baseline and change over time across groups by wearable device (ActiGraph, Modus). a** Self-entry Amyotrophic Lateral Sclerosis Functional Rating Scale-Revised (ALSFRS-RSE). **b** Rasch-built Overall ALS Disability Scale (ROADS). Participants are color coded and the color scheme is maintained across plots: lines represent participant-specific conditional means, points represent observed values. The thick black lines in **a**, **b** represent the population mean by wearable device: ActiGraph (solid line), Modus (dashed line).

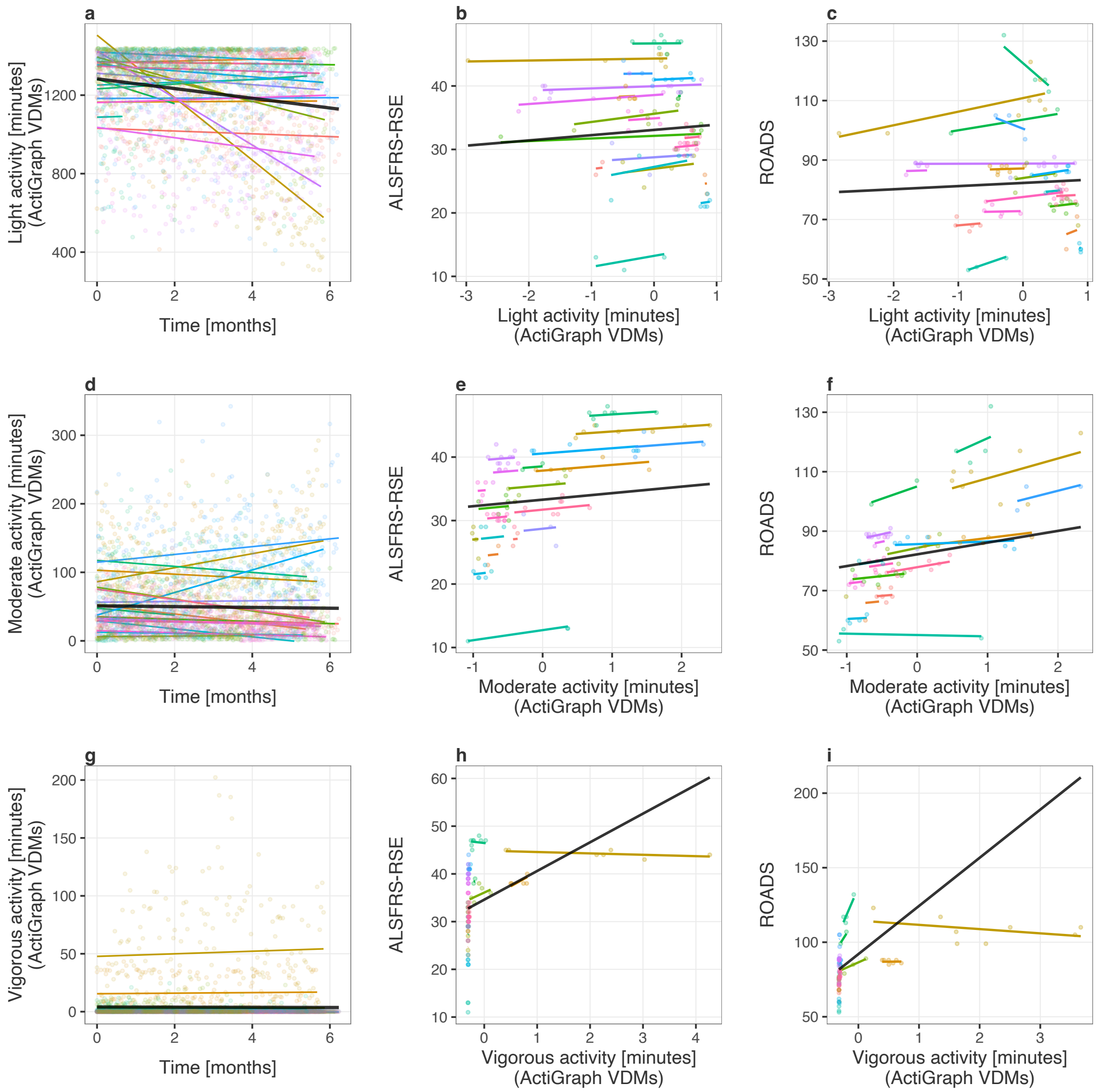

Supplementary Figure 2. **Wearable physical activity daily measure change over time and their association with the self-entry amyotrophic lateral sclerosis (ALS) functional rating scale-revised (ALSFRS-RSE) and the Rasch-built Overall ALS disability Scale (ROADS)**. Three different daily measures are represented in the figure, one per row. Column 1 (**a, d, g**) - baseline and monthly change in the daily measure. Column 2 (**b, e, h**) - daily measure association with ALSFRS-RSE total score. Column 3 (**c, f, i**) - daily measure association with the ROADS total score. The slopes in columns 2 and 3 represent the effect estimates for daily measures, standardized to have 0 means and unit standard deviations. In each plot, colored lines represent participant conditional mean values, and the color scheme is maintained across plots. Black lines represent population mean values.

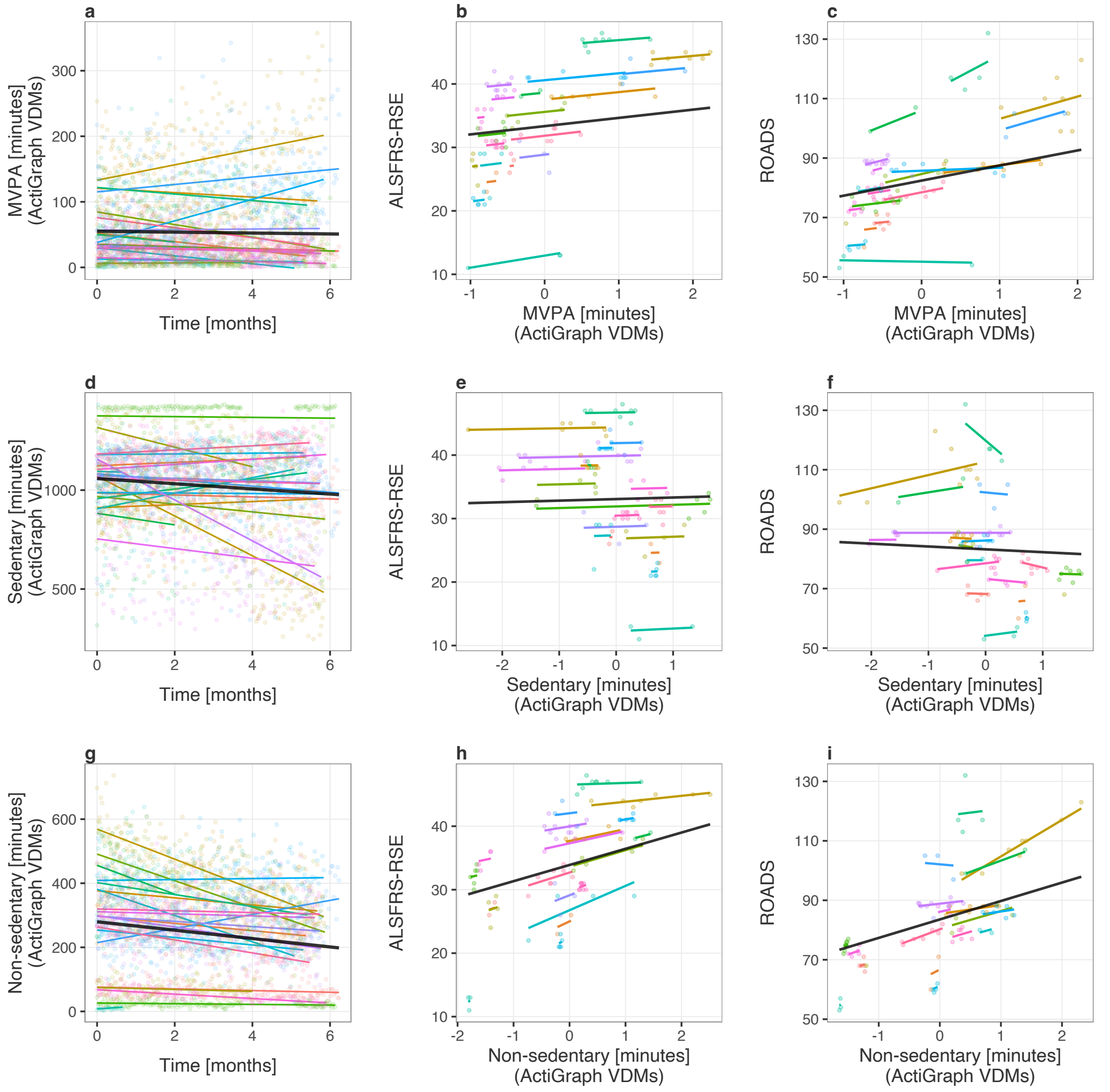

Supplementary Figure 3. **Wearable physical activity daily measure change over time and their association with the self-entry amyotrophic lateral sclerosis (ALS) functional rating scale-revised (ALSFRS-RSE) and the Rasch-built Overall ALS disability Scale (ROADS)**. Three different daily measures are represented in the figure, one per row. Column 1 (**a, d, g**) - baseline and monthly change in the daily measure. Column 2 (**b, e, h**) - daily measure association with ALSFRS-RSE total score. Column 3 (**c, f, i**) - daily measure association with the ROADS total score. The slopes in columns 2 and 3 represent the effect estimates for daily measures, standardized to have 0 means and unit standard deviations. In each plot, colored lines represent participant conditional mean values, and the color scheme is maintained across plots. Black lines represent population mean values.

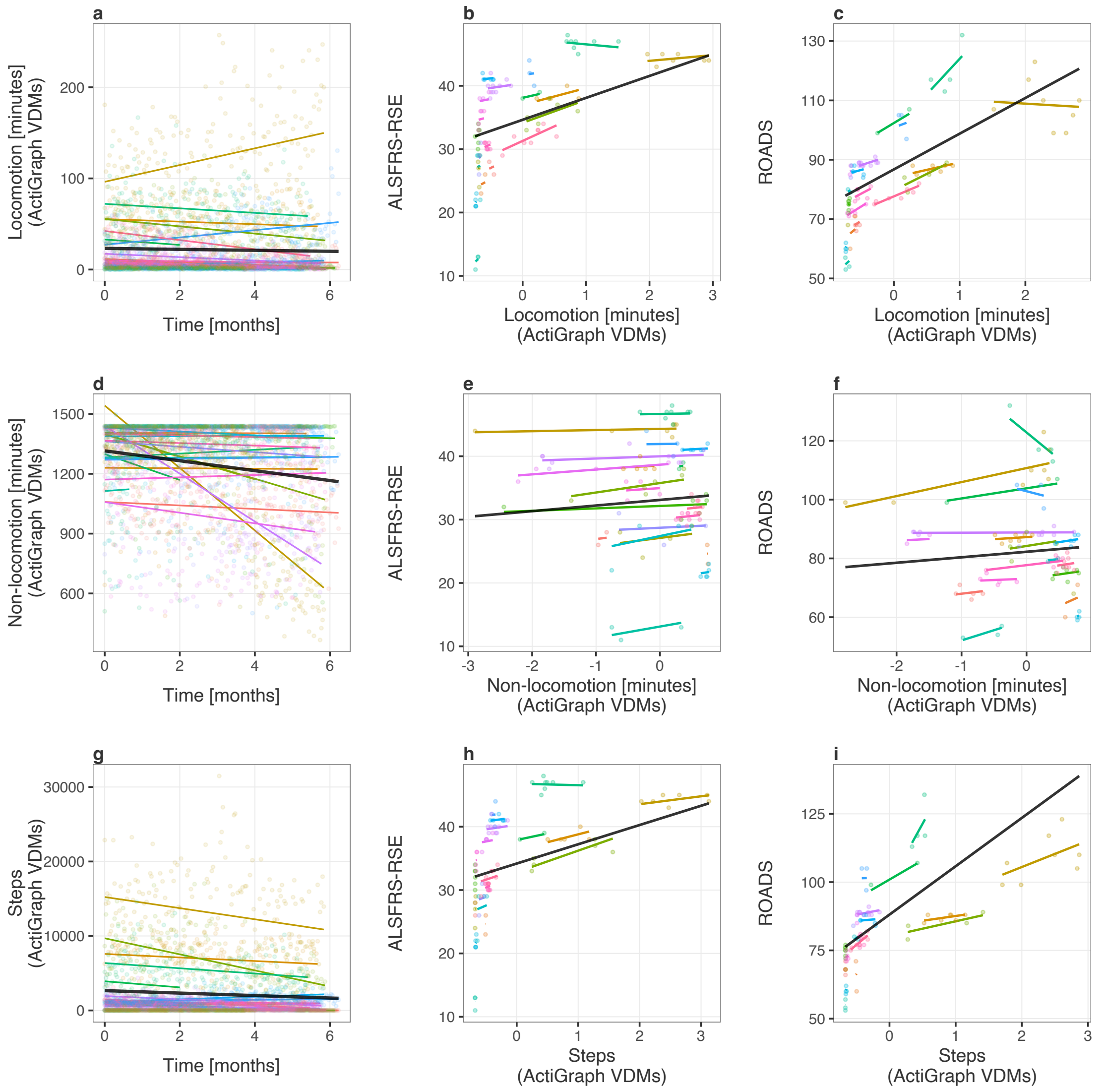

Supplementary Figure 4. **Wearable physical activity daily measure change over time and their association with the self-entry amyotrophic lateral sclerosis (ALS) functional rating scale-revised (ALSFRS-RSE) and the Rasch-built Overall ALS disability Scale (ROADS)**. Three different daily measures are represented in the figure, one per row. Column 1 (**a, d, g**) - baseline and monthly change in the daily measure. Column 2 (**b, e, h**) - daily measure association with ALSFRS-RSE total score. Column 3 (**c, f, i**) - daily measure association with the ROADS total score. The slopes in columns 2 and 3 represent the effect estimates for daily measures, standardized to have 0 means and unit standard deviations. In each plot, colored lines represent participant conditional mean values, and the color scheme is maintained across plots. Black lines represent population mean values.

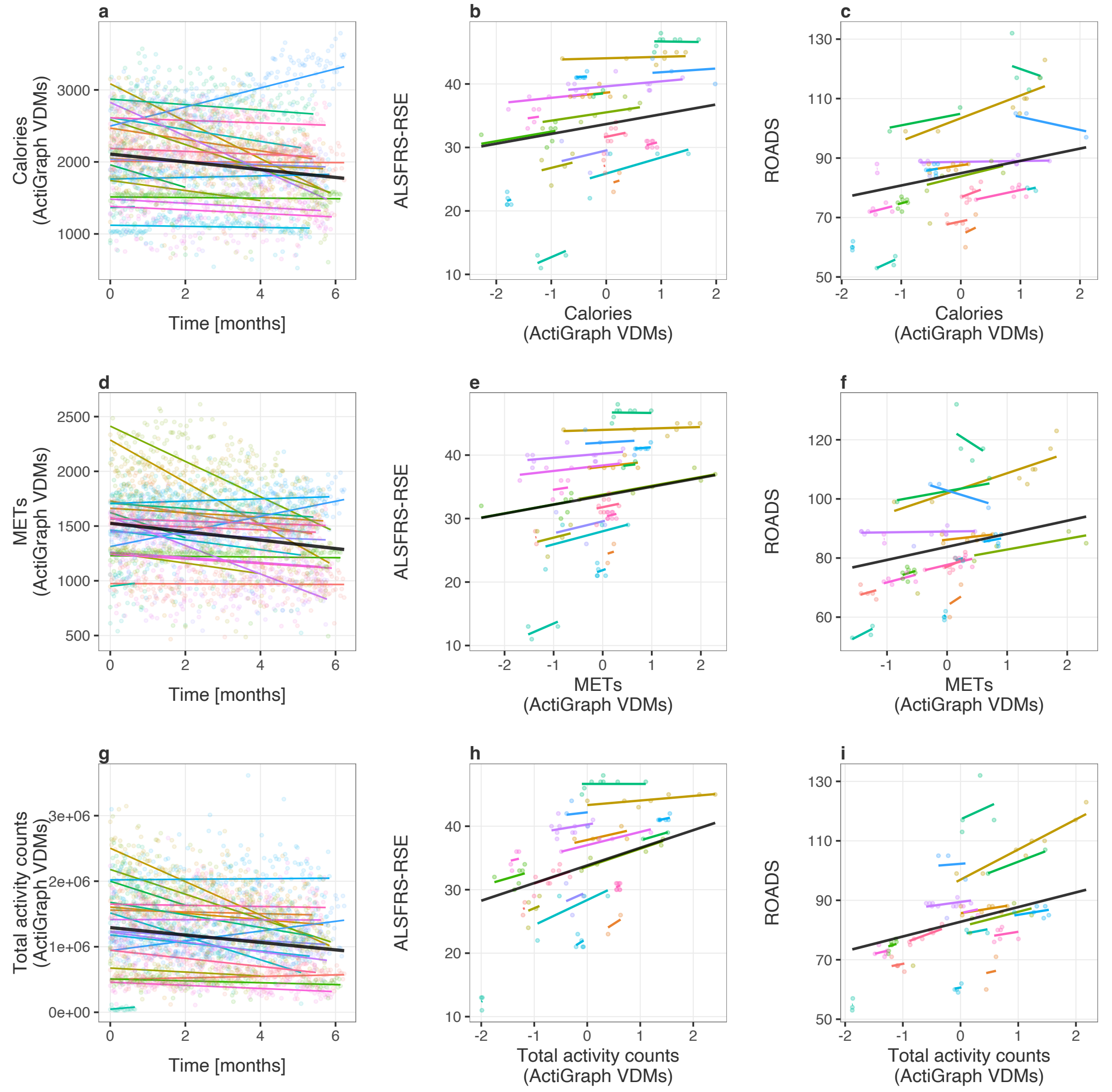

Supplementary Figure 5. **Wearable physical activity daily measure change over time and their association with the self-entry amyotrophic lateral sclerosis (ALS) functional rating scale-revised (ALSFRS-RSE) and the Rasch-built Overall ALS disability Scale (ROADS)**. Three different daily measures are represented in the figure, one per row. Column 1 (**a, d, g**) - baseline and monthly change in the daily measure. Column 2 (**b, e, h**) - daily measure association with ALSFRS-RSE total score. Column 3 (**c, f, i**) - daily measure association with the ROADS total score. The slopes in columns 2 and 3 represent the effect estimates for daily measures, standardized to have 0 means and unit standard deviations. In each plot, colored lines represent participant conditional mean values, and the color scheme is maintained across plots. Black lines represent population mean values.

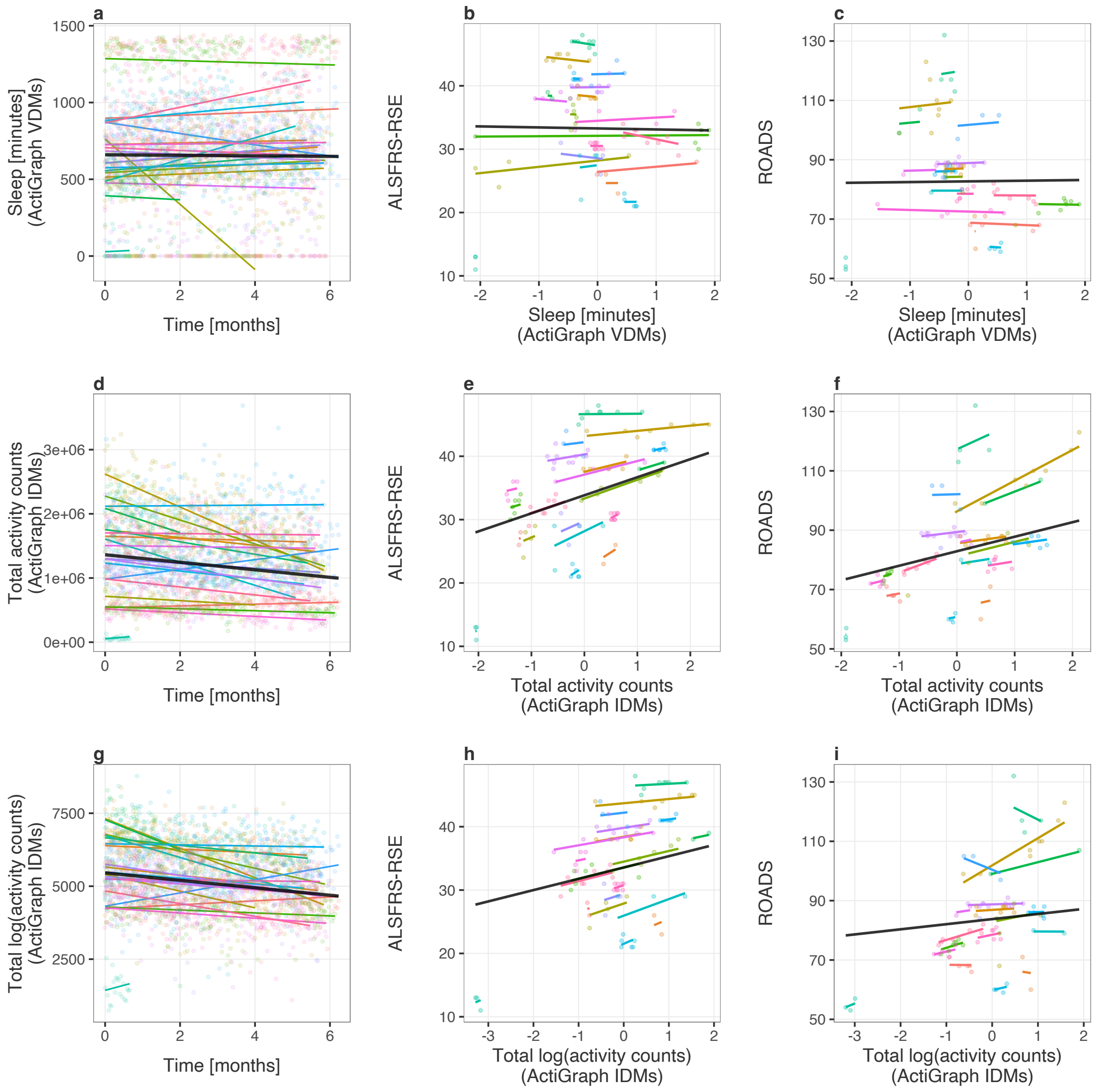

Supplementary Figure 6. **Wearable physical activity daily measure change over time and their association with the self-entry amyotrophic lateral sclerosis (ALS) functional rating scale-revised (ALSFRS-RSE) and the Rasch-built Overall ALS disability Scale (ROADS)**. Three different daily measures are represented in the figure, one per row. Column 1 (**a, d, g**) - baseline and monthly change in the daily measure. Column 2 (**b, e, h**) - daily measure association with ALSFRS-RSE total score. Column 3 (**c, f, i**) - daily measure association with the ROADS total score. The slopes in columns 2 and 3 represent the effect estimates for daily measures, standardized to have 0 means and unit standard deviations. In each plot, colored lines represent participant conditional mean values, and the color scheme is maintained across plots. Black lines represent population mean values.

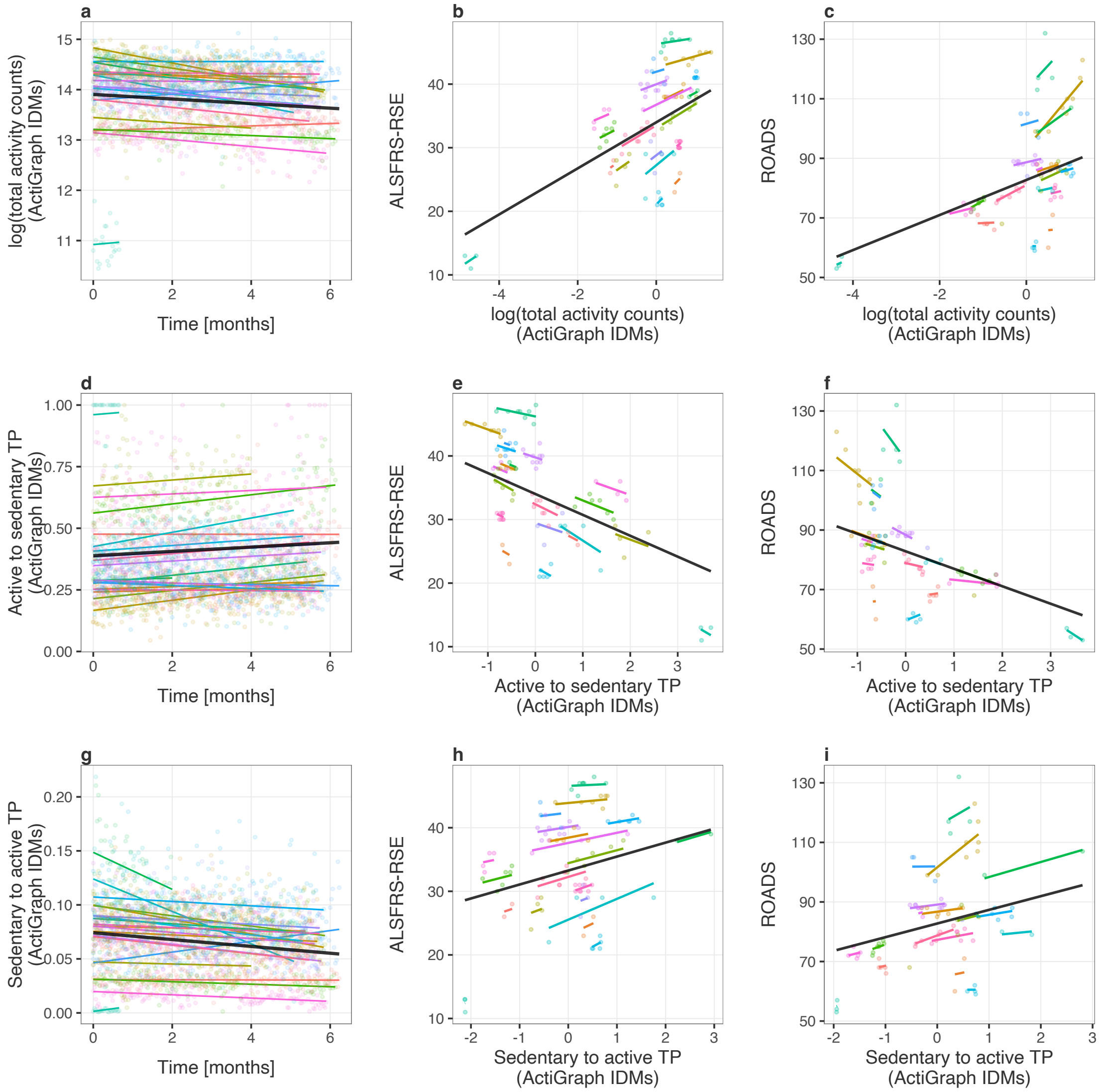

Supplementary Figure 7. **Wearable physical activity daily measure change over time and their association with the self-entry amyotrophic lateral sclerosis (ALS) functional rating scale-revised (ALSFRS-RSE) and the Rasch-built Overall ALS disability Scale (ROADS)**. Three different daily measures are represented in the figure, one per row. Column 1 (**a, d, g**) - baseline and monthly change in the daily measure. Column 2 (**b, e, h**) - daily measure association with ALSFRS-RSE total score. Column 3 (**c, f, i**) - daily measure association with the ROADS total score. The slopes in columns 2 and 3 represent the effect estimates for daily measures, standardized to have 0 means and unit standard deviations. In each plot, colored lines represent participant conditional mean values, and the color scheme is maintained across plots. Black lines represent population mean values.

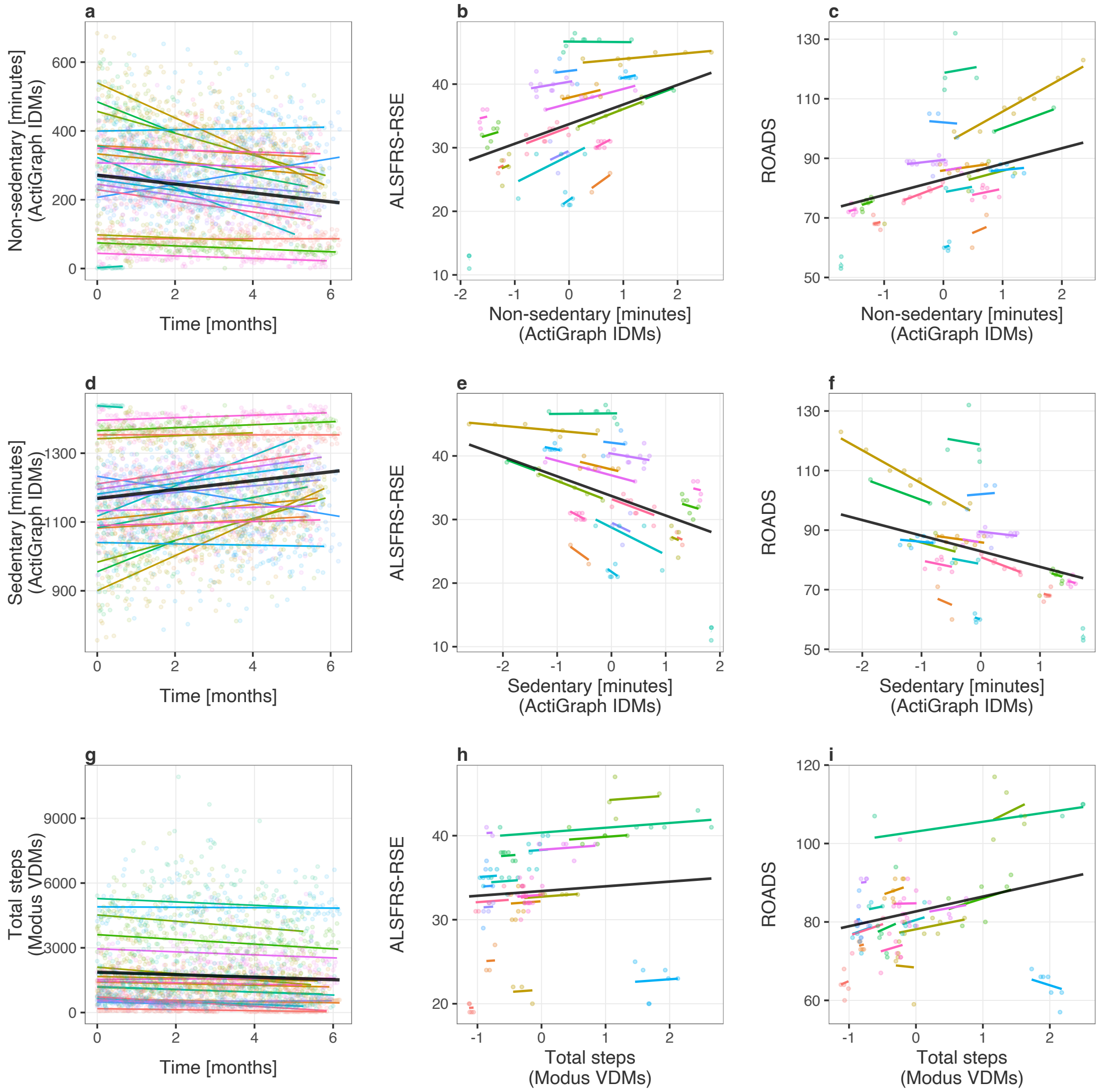

Supplementary Figure 8. **Wearable physical activity daily measure change over time and their association with the self-entry amyotrophic lateral sclerosis (ALS) functional rating scale-revised (ALSFRS-RSE) and the Rasch-built Overall ALS disability Scale (ROADS)**. Three different daily measures are represented in the figure, one per row. Column 1 (**a, d, g**) - baseline and monthly change in the daily measure. Column 2 (**b, e, h**) - daily measure association with ALSFRS-RSE total score. Column 3 (**c, f, i**) - daily measure association with the ROADS total score. The slopes in columns 2 and 3 represent the effect estimates for daily measures, standardized to have 0 means and unit standard deviations. In each plot, colored lines represent participant conditional mean values, and the color scheme is maintained across plots. Black lines represent population mean values.

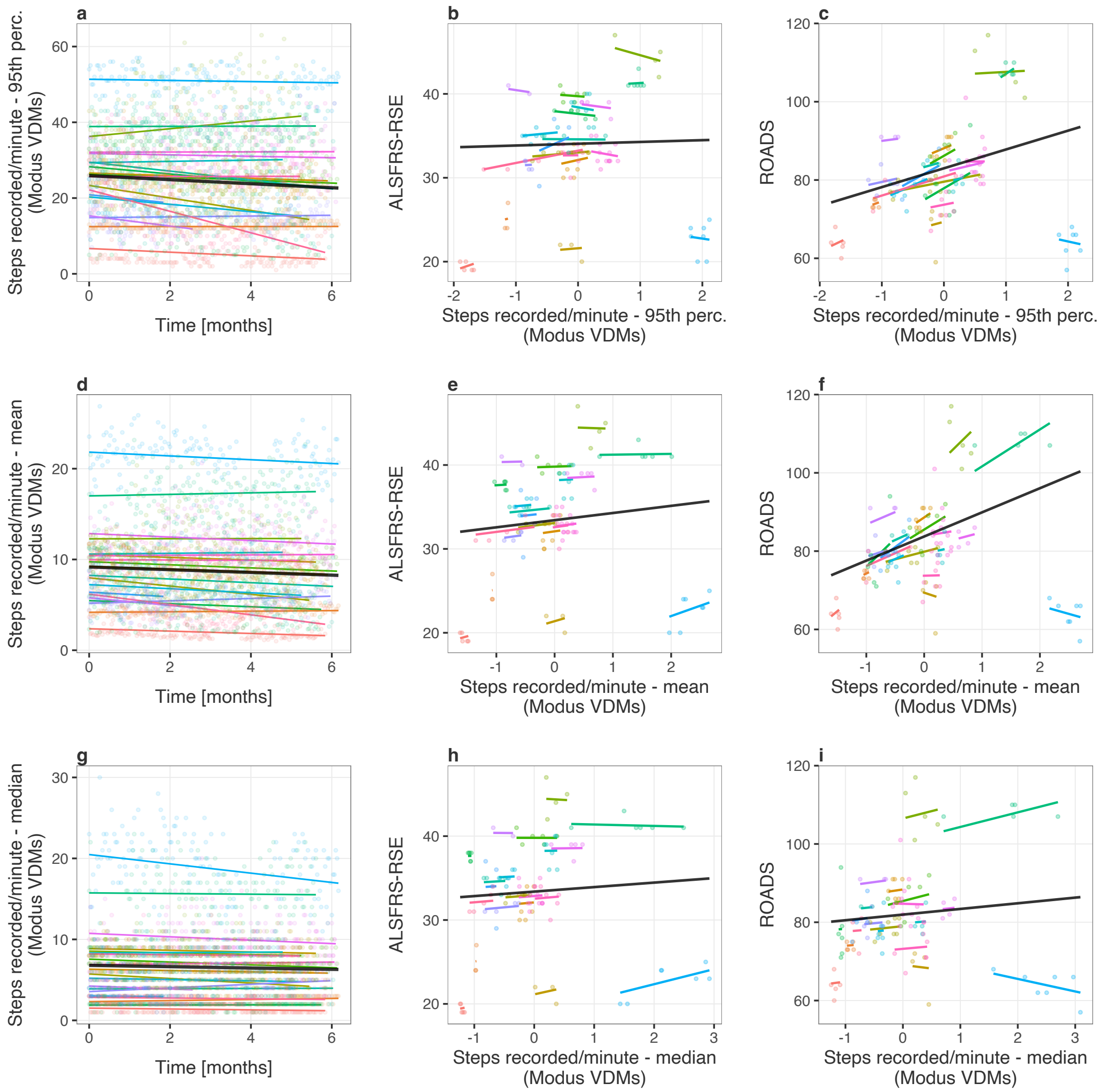

Supplementary Figure 9. **Wearable physical activity daily measure change over time and their association with the self-entry amyotrophic lateral sclerosis (ALS) functional rating scale-revised (ALSFRS-RSE) and the Rasch-built Overall ALS disability Scale (ROADS)**. Three different daily measures are represented in the figure, one per row. Column 1 (**a, d, g**) - baseline and monthly change in the daily measure. Column 2 (**b, e, h**) - daily measure association with ALSFRS-RSE total score. Column 3 (**c, f, i**) - daily measure association with the ROADS total score. The slopes in columns 2 and 3 represent the effect estimates for daily measures, standardized to have 0 means and unit standard deviations. In each plot, colored lines represent participant conditional mean values, and the color scheme is maintained across plots. Black lines represent population mean values.

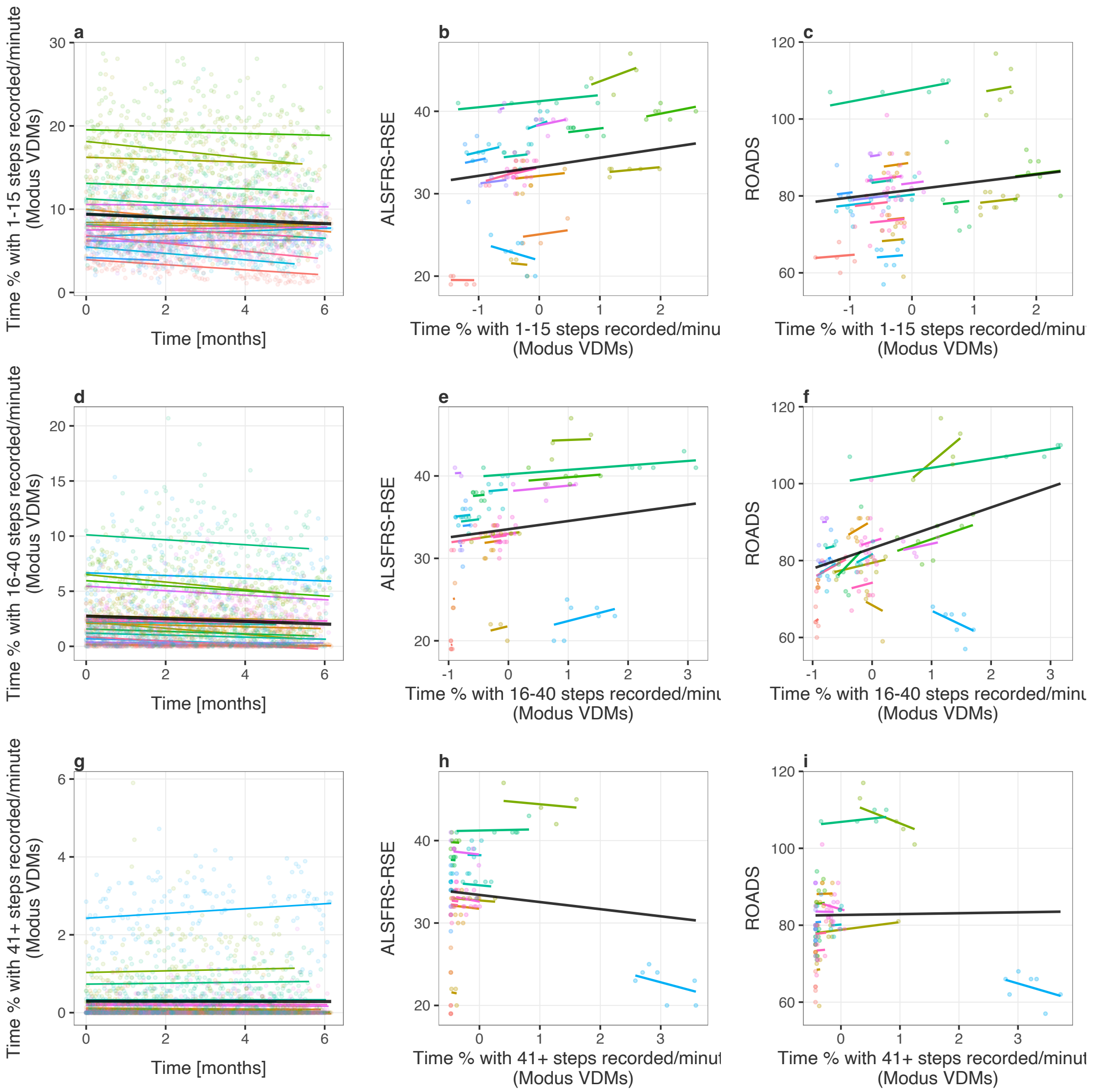

Supplementary Figure 10. **Wearable physical activity daily measure change over time and their association with the self-entry amyotrophic lateral sclerosis (ALS) functional rating scale-revised (ALSFRS-RSE) and the Rasch-built Overall ALS disability Scale (ROADS)**. Three different daily measures are represented in the figure, one per row. Column 1 (**a, d, g**) - baseline and monthly change in the daily measure. Column 2 (**b, e, h**) - daily measure association with ALSFRS-RSE total score. Column 3 (**c, f, i**) - daily measure association with the ROADS total score. The slopes in columns 2 and 3 represent the effect estimates for daily measures, standardized to have 0 means and unit standard deviations. In each plot, colored lines represent participant conditional mean values, and the color scheme is maintained across plots. Black lines represent population mean values.

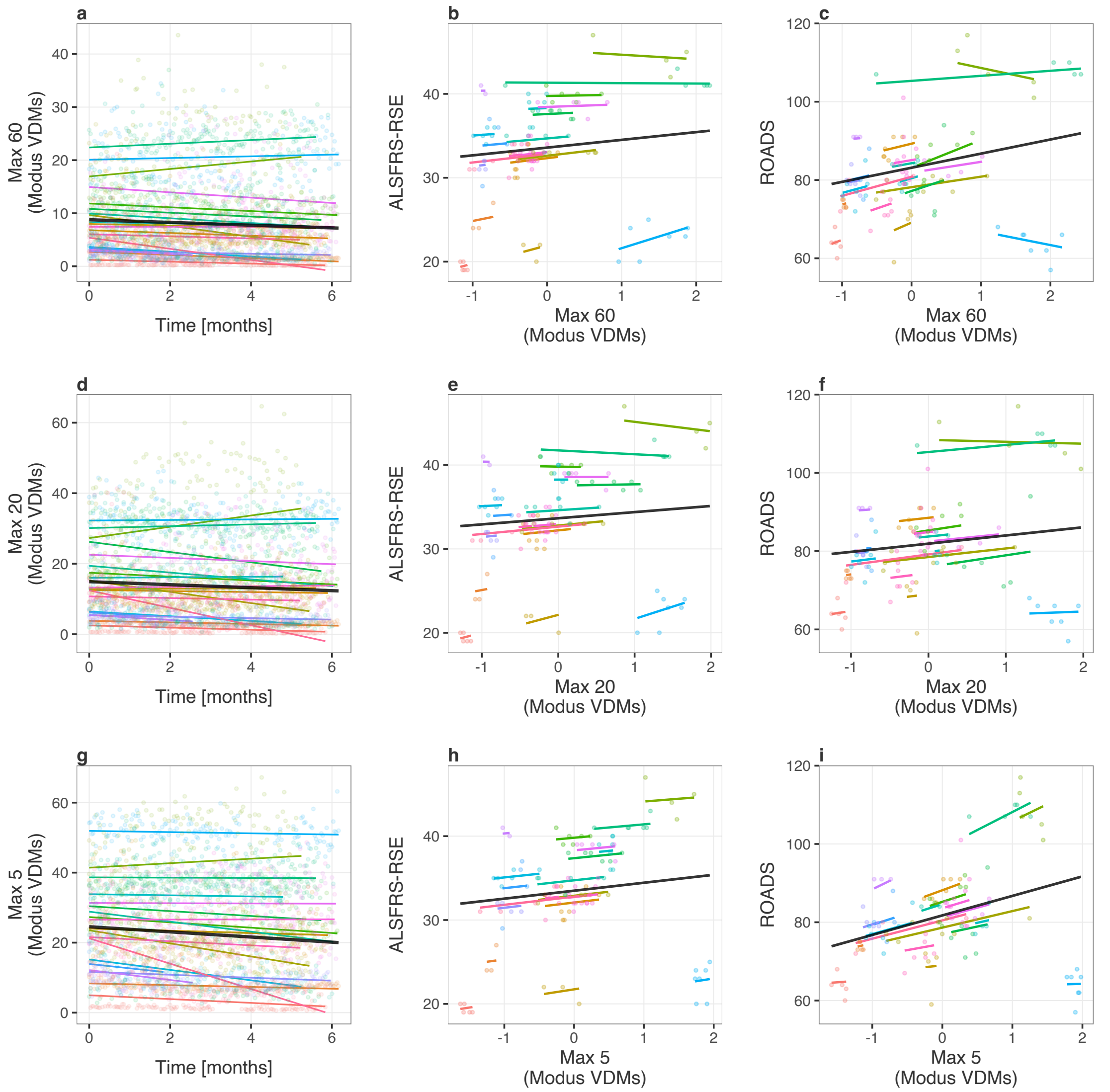

Supplementary Figure 11. **Wearable physical activity daily measure change over time and their association with the self-entry amyotrophic lateral sclerosis (ALS) functional rating scale-revised (ALSFRS-RSE) and the Rasch-built Overall ALS disability Scale (ROADS)**. Three different daily measures are represented in the figure, one per row. Column 1 (**a, d, g**) - baseline and monthly change in the daily measure. Column 2 (**b, e, h**) - daily measure association with ALSFRS-RSE total score. Column 3 (**c, f, i**) - daily measure association with the ROADS total score. The slopes in columns 2 and 3 represent the effect estimates for daily measures, standardized to have 0 means and unit standard deviations. In each plot, colored lines represent participant conditional mean values, and the color scheme is maintained across plots. Black lines represent population mean values.

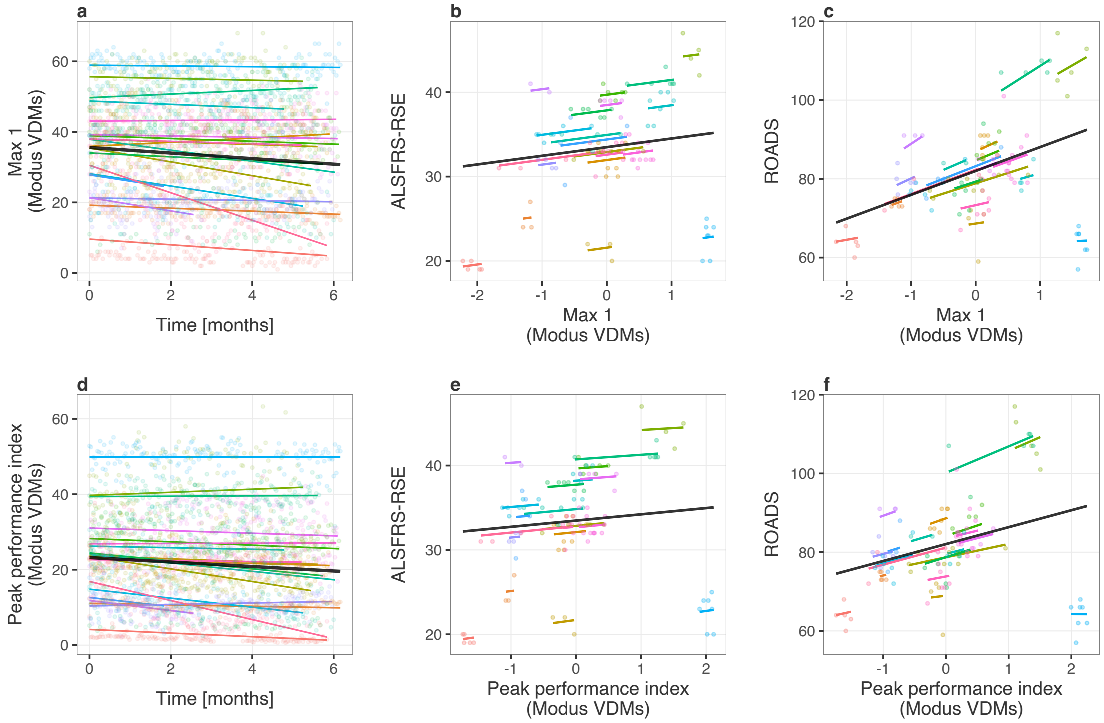

Supplementary Figure 12. **Wearable physical activity daily measure change over time and their association with the self-entry amyotrophic lateral sclerosis (ALS) functional rating scale-revised (ALSFRS-RSE) and the Rasch-built overall ALS disability Scale (ROADS)**. Two different daily measures are represented in the figure, one per row. Column 1 (**a, d**) - baseline and monthly change in the daily measure. Column 2 (**b, e**) - daily measure association with ALSFRS-RSE total score. Column 3 (**c, f**) - daily measure association with the ROADS total score. The slopes in columns 2 and 3 represent the effect estimates for daily measures, standardized to have 0 means and unit standard deviations. In each plot, colored lines represent participant conditional mean values, and the color scheme is maintained across plots. Black lines represent population mean values.

Supplementary Table 1

LMMs quantifying population average baselines and monthly change in ALSFRS-RSE, ALSFRS-R and ROADS.

Model	Outcome	Term	Term est. [95% CI] (p-val.)	R2m	R2c.
1	ALSFRS-RSE	Intercept	34.346 [31.950, 36.736] (0.000)	0.013	0.98
		Time	-0.475 [-0.627, -0.325] (0.000)		
2	ROADS	Intercept	84.906 [79.904, 89.896] (0.000)	0.025	0.954
		Time [months]	-1.260 [-1.712, -0.806] (0.000)		
3	ALSFRS-R	Intercept	31.576 [29.112, 34.039] (0.000)	0.014	0.942
		Time [months]	-0.369 [-0.623, -0.113] (0.007)		
4	(ALSFRS-RSE, ALSFRS-R)	Intercept	34.422 [32.033, 36.808] (0.000)	0.046	0.957
		Time [months]	-0.473 [-0.702, -0.247] (0.000)		
		in-clinic	-2.866 [-3.470, -2.257] (0.000)		
		Time [months]:in-clinic	0.057 [-0.122, 0.234] (0.535)		
5	ALSFRS-RSE	Intercept	34.597 [31.219, 37.961] (0.000)	0.013	0.981
		Time [months]	-0.595 [-0.805, -0.388] (0.000)		
		Modus	-0.484 [-5.241, 4.287] (0.844)		
		Time [months]:Modus	0.236 [-0.054, 0.528] (0.115)		
6	ROADS	Intercept	85.547 [78.453, 92.583] (0.000)	0.025	0.956
		Time [months]	-1.381 [-2.035, -0.710] (0.000)		
		Modus	-1.246 [-11.191, 8.766] (0.808)		
		Time [months]:Modus	0.223 [-0.701, 1.122] (0.632)		

ALSFRS-R -- staff-administered amyotrophic lateral sclerosis functional rating scale-revised; ALSFRS-RSE -- self-entry ALSFRS-R; CI -- estimate's confidence interval obtained from LMM estimation; est. -- estimate obtained from LMM estimation; LMM – mixed effects model; Mod. no. -- LMM model number; R2c -- LMM conditional coefficient of determination; R2m -- LMM marginal coefficient of determination; p.-val. -- estimate's p-value obtained from LMM estimation; ROADS -- Rasch-built Overall ALS Disability Scale

Supplementary Table 2

Association between wearable-derived daily PA measures and ALSFRS-RSE total score

Model no.	Device group	Data set	Daily measure	Slope est.* [95% CI] (p-val.)	R2m	R2c
1	AG	VDMs	Light activity [minutes]	0.828 [-1.119, 2.775] (0.178)	0.006	0.975
2	AG	VDMs	Moderate activity [minutes]	1.028 [0.254, 1.802] (0.010)	0.011	0.97
3	AG	VDMs	Vigorous activity [minutes]	6.009 [3.433, 8.585] (0.002)	0.226	0.976
4	AG	VDMs	MVPA [minutes]	1.302 [0.319, 2.285] (0.010)	0.018	0.969
5	AG	VDMs	Sedentary [minutes]	0.246 [-0.333, 0.824] (0.400)	0.001	0.972
6	AG	VDMs	Non-sedentary [minutes]	2.553 [1.687, 3.420] (0.000)	0.103	0.974
7	AG	VDMs	Locomotion [minutes]	3.462 [2.237, 4.687] (0.000)	0.148	0.969
8	AG	VDMs	Non-locomotion [minutes]	0.886 [-0.409, 2.181] (0.114)	0.006	0.976
9	AG	VDMs	Steps	3.040 [1.950, 4.130] (0.000)	0.123	0.970
10	AG	VDMs	Calories	1.543 [0.774, 2.313] (0.000)	0.032	0.973
11	AG	VDMs	METs	1.414 [0.744, 2.083] (0.000)	0.025	0.972
12	AG	VDMs	Total activity counts	2.778 [1.837, 3.720] (0.000)	0.116	0.976
13	AG	VDMs	Sleep [minutes]	-0.159 [-3.577, 3.259] (0.831)	0.000	0.970
14	AG	IDMs	Total activity counts	2.850 [1.883, 3.817] (0.000)	0.124	0.974
15	AG	IDMs	Total log(activity counts)	1.784 [1.028, 2.541] (0.000)	0.047	0.971
16	AG	IDMs	log(total activity counts)	3.621 [2.386, 4.857] (0.000)	0.237	0.977
17	AG	IDMs	Active to sedentary TP	-3.286 [-5.036, -1.536] (0.003)	0.198	0.971
18	AG	IDMs	Sedentary to active TP	2.197 [1.293, 3.100] (0.000)	0.061	0.977
19	AG	IDMs	Non-sedentary [minutes]	3.082 [2.042, 4.121] (0.000)	0.134	0.977
20	AG	IDMs	Sedentary [minutes]	-3.082 [-4.121, -2.042] (0.000)	0.134	0.977
21	M	VDMs	Total steps	0.564 [-0.315, 1.443] (0.201)	0.005	0.958
22	M	VDMs	Steps recorded/minute - 95th perc.	0.213 [-1.832, 2.258] (0.810)	0.001	0.961
23	M	VDMs	Steps recorded/minute - mean	0.854 [-0.431, 2.139] (0.191)	0.011	0.964
24	M	VDMs	Steps recorded/minute - median	0.536 [-0.438, 1.511] (0.277)	0.005	0.964
25	M	VDMs	Time % with 1-15 steps recorded/minute	1.097 [-0.522, 2.716] (0.159)	0.018	0.964
26	M	VDMs	Time % with 16-40 steps recorded/minute	0.993 [0.274, 1.711] (0.007)	0.016	0.960
27	M	VDMs	Time % with 41+ steps recorded/minute	-0.863 [-3.117, 1.390] (0.363)	0.012	0.961
28	M	VDMs	Max 60	0.923 [0.137, 1.709] (0.022)	0.011	0.964
29	M	VDMs	Max 20	0.731 [-0.252, 1.715] (0.142)	0.007	0.964
30	M	VDMs	Max 5	0.949 [-0.149, 2.048] (0.090)	0.013	0.958
31	M	VDMs	Max 1	1.025 [-0.008, 2.058] (0.052)	0.017	0.957
32	M	VDMs	Peak performance index	0.741 [-0.384, 1.866] (0.195)	0.009	0.958

AG -- ActiGraph device; ALSFRS-RSE -- self-entry amyotrophic lateral sclerosis functional rating scale-revised; IDMs -- investigator-derived daily measures; CI -- estimate's confidence interval obtained from LMM estimation; est. -- estimate obtained from LMM estimation; Model no. – model number: ordering index of a LMM fit, assigned to a particular daily measure; R2c -- LMM conditional coefficient of determination; R2m -- LMM marginal coefficient of determination; M -- Modus device; Max (60,20,5,1) – maximum consecutive cadence during (60,20,5,1) minutes; PA -- physical activity; PPI -- peak performance index (mean cadence of the day's most intensive, non-contiguous 30 minutes); VDMs -- vendor-provided daily measures

* Daily measure slopes have been standardized to have means of zero and units in standard deviations.

Supplementary Table 3

Association between wearable-derived daily PA measures and ROADS score.

Model no.	Device group	Data set	Daily measure	Slope est.* [95% CI] (p-val.)	R2m	R2c
1	AG	VDMs	Light activity [minutes]	1.066 [-4.157, 6.288] (0.663)	0.002	0.977
2	AG	VDMs	Moderate activity [minutes]	3.947 [0.214, 7.680] (0.041)	0.044	0.947
3	AG	VDMs	Vigorous activity [minutes]	32.32 [-28.05, 92.68] (0.211)	0.269	0.995
4	AG	VDMs	MVPA [minutes]	5.074 [0.469, 9.680] (0.035)	0.077	0.944
5	AG	VDMs	Sedentary [minutes]	-0.954 [-6.155, 4.247] (0.690)	0.002	0.968
6	AG	VDMs	Non-sedentary [minutes]	6.202 [1.932, 10.47] (0.009)	0.145	0.969
7	AG	VDMs	Locomotion [minutes]	12.04 [4.131, 19.94] (0.007)	0.346	0.963
8	AG	VDMs	Non-locomotion [minutes]	1.869 [-3.376, 7.114] (0.447)	0.006	0.978
9	AG	VDMs	Steps	17.70 [4.817, 30.59] (0.013)	0.447	0.980
10	AG	VDMs	Calories	4.138 [0.053, 8.223] (0.048)	0.047	0.974
11	AG	VDMs	METs	4.422 [-0.518, 9.363] (0.072)	0.049	0.973
12	AG	VDMs	Total activity counts	4.945 [1.081, 8.809] (0.018)	0.092	0.967
13	AG	VDMs	Sleep [minutes]	0.233 [-2.664, 3.130] (0.872)	0.000	0.952
14	AG	IDMs	Total activity counts	4.891 [0.899, 8.883] (0.021)	0.092	0.966
15	AG	IDMs	Total log(activity counts)	1.720 [-2.231, 5.671] (0.360)	0.009	0.972
16	AG	IDMs	log(total activity counts)	5.888 [1.065, 10.71] (0.021)	0.157	0.967
17	AG	IDMs	Active to sedentary TP	-5.872 [-9.531, -2.214] (0.004)	0.150	0.948
18	AG	IDMs	Sedentary to active TP	4.588 [0.128, 9.048] (0.045)	0.070	0.957
19	AG	IDMs	Non-sedentary [minutes]	5.235 [1.275, 9.195] (0.014)	0.101	0.969
20	AG	IDMs	Sedentary [minutes]	-5.235 [-9.195, -1.275] (0.014)	0.101	0.969
21	M	VDMs	Total steps	3.791 [-2.403, 9.985] (0.188)	0.083	0.869
22	M	VDMs	Steps recorded/minute - 95th perc.	4.810 [-0.445, 10.07] (0.068)	0.113	0.877
23	M	VDMs	Steps recorded/minute - mean	6.167 [0.719, 11.61] (0.030)	0.195	0.883
24	M	VDMs	Steps recorded/minute - median	1.445 [-3.753, 6.643] (0.536)	0.013	0.856
25	M	VDMs	Time % with 1-15 steps recorded/minute	1.992 [-1.330, 5.314] (0.236)	0.022	0.860
26	M	VDMs	Time % with 16-40 steps recorded/minute	5.309 [-1.930, 12.55] (0.126)	0.125	0.907
27	M	VDMs	Time % with 41+ steps recorded/minute	0.231 [-9.796, 10.26] (0.943)	0.000	0.893
28	M	VDMs	Max 60	3.609 [-1.051, 8.269] (0.112)	0.059	0.894
29	M	VDMs	Max 20	2.096 [-1.929, 6.121] (0.228)	0.020	0.873
30	M	VDMs	Max 5	4.986 [1.805, 8.167] (0.003)	0.134	0.857
31	M	VDMs	Max 1	6.062 [3.141, 8.984] (0.000)	0.201	0.873
32	M	VDMs	Peak performance index	4.298 [-1.822, 10.42] (0.105)	0.114	0.849

AG -- ActiGraph device; IDMs -- investigator-derived daily measures; CI -- estimate's confidence interval obtained from LMM estimation; est. -- estimate obtained from LMM estimation; Model no. -- model number: ordering index of a LMM fit, assigned to a particular daily measure; R2c -- LMM conditional coefficient of determination; R2m -- LMM marginal coefficient of determination; M -- Modus device; Max (60,20,5,1) – maximum consecutive cadence during (60,20,5,1) minutes; PA – physical activity; PPI -- peak performance index (mean cadence of the day's most intensive, non-contiguous 30 minutes); ROADS -- Rasch-built Overall ALS Disability Scale; VDMs -- vendor-provided daily measures

* Daily measure slopes have been standardized to have means of zero and units in standard deviations.

Supplementary Table 4

Average baseline and monthly change in investigator-derived total activity counts across 9 different data collection scenarios

Time span of data included	No. of data points	Baseline est. [95% CI]	Baseline est. % change	Monthly change est. [95% CI] (p-val.)	Monthly change est. % change
All wD	2821	1362438 [1059453, 1665100]		-58631 [-99870, -16735] (0.012)	
2wD+2wB	1480	1365618 [1062850, 1668039]	0.2%	-60004 [-99344, -20021] (0.007)	2.3%
2wD+4wB	1030	1369841 [1063604, 1675745]	0.5%	-60904 [-101906, -19606] (0.009)	3.9%
2wD+6wB	839	1370795 [1067516, 1673669]	0.6%	-59797 [-100155, -19086] (0.009)	2.0%
2wD+8wB	690	1378268 [1071597, 1684976]	1.2%	-50122 [-94237, -4581] (0.038)	-14.5%
1wD+2wB	982	1383447 [1081313, 1685141]	1.5%	-64690 [-106144, -22614] (0.006)	10.3%
1wD+4wB	616	1378843 [1074813, 1682415]	1.2%	-53240 [-96037, -10231] (0.024)	-9.2%
1wD+6wB	446	1362648 [1058805, 1666175]	0.0%	-73018 [-117645, -27579] (0.005)	24.5%
1wD+8wB	357	1360927 [1061328, 1660141]	-0.1%	-52865 [-100359, -3977] (0.041)	-9.8%

% change – the percent change in point estimate value as compared with the estimate obtained with the use of all available data ("All wD"); CI -- estimate's confidence interval obtained from LMM estimation; est. -- estimate obtained from LMM estimation; No. -- number; p-val. -- estimate's p-value obtained from LMM estimation; wD -- week(s) of data included in the analysis; wB -- weeks of data withheld from the analysis (“break in wearable data collection")
