# Supplementary material for: Wearable device and smartphone data quantify ALS progression and may provide novel outcome measures
Source: NPJ Digit Med. 2023 Mar 6;6:34. doi: 10.1038/s41746-023-00778-y (PMC9987377; doi:10.1038/s41746-023-00778-y)
Supplement: Supplementary file 2 — REPORTING SUMMARY [file 41746_2023_778_MOESM2_ESM.pdf]

nature portfolio

Corresponding author(s): DBPR NPJDIGITALMED-05258R

Last updated by author(s): 2023-01-14

Reporting Summary

Nature Portfolio wishes to improve the reproducibility of the work that we publish. This form provides structure for consistency and transparency in reporting. For further information on Nature Portfolio policies, see our Editorial Policies and the Editorial Policy Checklist.

Statistics

For all statistical analyses, confirm that the following items are present in the figure legend, table legend, main text, or Methods section.

n/a	Confirmed	
☐	☒	The exact sample size (*n*) for each experimental group/condition, given as a discrete number and unit of measurement
☐	☒	A statement on whether measurements were taken from distinct samples or whether the same sample was measured repeatedly
☐	☒	The statistical test(s) used AND whether they are one- or two-sided<br>*Only common tests should be described solely by name; describe more complex techniques in the Methods section.*
☐	☒	A description of all covariates tested
☐	☒	A description of any assumptions or corrections, such as tests of normality and adjustment for multiple comparisons
☐	☒	A full description of the statistical parameters including central tendency (e.g. means) or other basic estimates (e.g. regression coefficient) AND variation (e.g. standard deviation) or associated estimates of uncertainty (e.g. confidence intervals)
☐	☒	For null hypothesis testing, the test statistic (e.g. *F*, *t*, *r*) with confidence intervals, effect sizes, degrees of freedom and *P* value noted<br>*Give P values as exact values whenever suitable.*
☒	☐	For Bayesian analysis, information on the choice of priors and Markov chain Monte Carlo settings
☒	☐	For hierarchical and complex designs, identification of the appropriate level for tests and full reporting of outcomes
☐	☒	Estimates of effect sizes (e.g. Cohen's *d*, Pearson's *r*), indicating how they were calculated

Our web collection on statistics for biologists contains articles on many of the points above.

Software and code

Policy information about availability of computer code

Data collection	*Provide a description of all commercial, open source and custom code used to collect the data in this study, specifying the version used OR state that no software was used.* *see bottom of page**
Data analysis	All analyses were performed using R software (version 4.2.0; The R Project). The code for all data preprocessing and data analysis is available here, https://github.com/onnela-lab/als-wearables).

For manuscripts utilizing custom algorithms or software that are central to the research but not yet described in published literature, software must be made available to editors and reviewers. We strongly encourage code deposition in a community repository (e.g. GitHub). See the Nature Portfolio guidelines for submitting code & software for further information.

Data

Policy information about availability of data

All manuscripts must include a data availability statement. This statement should provide the following information, where applicable:

- Accession codes, unique identifiers, or web links for publicly available datasets
- A description of any restrictions on data availability
- For clinical datasets or third party data, please ensure that the statement adheres to our policy

Provide your data availability statement here.

Human research participants

Policy information about studies involving human research participants and Sex and Gender in Research.

Reporting on sex and gender	In our Table 1, we report the percentage of participants who self-report as either male or female sex; however, we did not collect information on gender. We did not compare groups based on sex given our sample size, the heterogeneity of the disease, and because this study is focused on understanding the feasibility of remotely collected patient-report outcome measures and physical function. Consequently, we did not feel we would be able to make meaningful statistical comparisons based on sex.
Population characteristics	The mean age was 62 years, all participants had a confirmed diagnosis of ALS.
Recruitment	The study was advertised on ALS social media accounts, an institutional research recruitment website, and to patients attending the ALS multidisciplinary clinic. Due to the convenience sample nature of recruitment, we likely are undersampling participants who are lack internet access and technological proficiency. The observational nature also means that this population is likely slower progressing than those who engage in interventional trials. Because we were examining the ability of technology to measure functional decline, results would be less robust than they otherwise would be.
Ethics oversight	Massachusetts General Brigham (MGB) Institutional Review Board (IRB)

Note that full information on the approval of the study protocol must also be provided in the manuscript.

Field-specific reporting

Please select the one below that is the best fit for your research. If you are not sure, read the appropriate sections before making your selection.

[X] Life sciences   [ ] Behavioural & social sciences   [ ] Ecological, evolutionary & environmental sciences

For a reference copy of the document with all sections, see nature.com/documents/nr-reporting-summary-flat.pdf

Life sciences study design

All studies must disclose on these points even when the disclosure is negative.

Sample size	Because this was a feasibility study, sample size was not predetermined. We enrolled the full number of participants for which funding allowed.
Data exclusions	see below*
Replication	This was a longitudinal 6-month study examining daily physical activity measures using two wearable devices and collecting patient reported outcome measures via personal smartphone survey administration. Replication would require repeating the study.
Randomization	Participants independently chose in an even split per their preference whether to participate in the Actigraph vs Modus arm of the study. Participants were compared to themselves over time as measuring functional decline was the aim of the study.
Blinding	Investigators were blind to participant identity during data/statistical analysis

Data exclusions*
Participants who did not complete at least two of each of the functional rating scale surveys were excluded n = 6. This was not pre-specified. This was because we were studying functional decline over time, and without a second time point, we could not assess change over time. For examining the wearable device derived metrics, only valid days were used, defined as days with at least 8, not necessarily consecutive, "valid hours." Due to device differences, valid hours were defined uniquely for each wearable. For Actigraph, a valid hour was defined as 60 consecutive minutes without missing data and vendor-provided wear status indicating device wear. For Modus, a valid hour was one with at least one step logged. These were chosen based on device measurement capabilities, sensitivity to detect movement, and what is standard practice in the literature.
Replication
